# Depression and anxiety in health human resources during the first COVID-19 wave in northern Peru: a multicenter study

**DOI:** 10.3389/fpsyt.2025.1616381

**Published:** 2025-12-12

**Authors:** Mario J. Valladares-Garrido, Carlos Culquichicon, Milagritos Sánchez Reto, Danai Valladares-Garrido, Víctor J. Vera-Ponce, César J. Pereira-Victorio, Virgilio E. Failoc-Rojas, J. Pierre Zila-Velasque, Cristian Díaz-Vélez, Wilde Lavado Acuña

**Affiliations:** 1 Escuela de Medicina Humana, Universidad Señor de Sipán, Chiclayo, Peru; 2 University of Washington, School of Public Health, Seatle, WA, United States; 3 Facultad de Ciencias de la Salud, Universidad Nacional de Piura, Piura, Peru; 4 Facultad de Medicina, Universidad Cesar Vallejo, Piura, Peru; 5 Unidad de Salud Ocupacional, Hospital de Apoyo II Santa Rosa, Piura, Peru; 6 EpiHealth Research Center for Epidemiology and Public Health, Lima, Peru; 7 Instituto de Investigación de Enfermedades Tropicales, Universidad Nacional Toribio Rodríguez de Mendoza de Amazonas (UNTRM), Amazonas, Peru; 8 Facultad de Medicina (FAMED), Universidad Nacional Toribio Rodríguez de Mendoza de Amazonas (UNTRM), Amazonas, Peru; 9 Facultad de Medicina, Universidad Continental, Lima, Peru; 10 Unidad de Investigación para Generación y Síntesis de Evidencia en Salud, Universidad San Ignacio de Loyola, Lima, Peru; 11 Red Latinoamericana de Medicina en la Altitud e Investigación (REDLAMAI), Pasco, Peru; 12 Instituto de Evaluación de Tecnologías en Salud e Investigación - IETSI, EsSalud, Lima, Peru; 13 Escuela de Posgrado, Facultad de Ciencias de la Salud, Universidad Científica del Sur, Lima, Peru; 14 Facultad de Medicina, Universidad de San Martín de Porres, Lima, Peru

**Keywords:** depression, anxiety, mental health, Peru, COVID-19

## Abstract

**Background:**

Although global evidence demonstrates a clear mental health impact of COVID-19 on healthcare workers, data from Latin American settings, particularly during the first pandemic wave and within social security hospital systems, remain limited and heterogeneous. The objective was to determine the prevalence and factors associated with depression and anxiety in health human resources of three hospitals of the Social Security of Piura and Lambayeque, during the first pandemic wave of COVID-19.

**Methods:**

Cross-sectional analytical study in which anxiety and depression, and their association with resilience, insomnia, physical activity, eating disorder, tobacco and alcohol consumption, Burnout Syndrome and physical, psychosocial, occupational and personal health variables were evaluated. Multivariate analyses were used to estimate prevalence ratios (PR) and generalized linear models (GLM) to identify association between variables.

**Results:**

Of 182 health care workers, the prevalence of depression and anxiety was 42.9% and 50.6%, respectively. The factors associated with depression were being diabetic (PR: 1.41), mistreatment (PR: 1.35), moderate concern about working in a COVID environment (PR: 1.23), much/extreme concern about working in a COVID environment (PR: 1.23), much/extreme concern about being marginalized by the surrounding environment (PR: 2.00), insomnia (PR: 1.62) and burnout syndrome (PR: 1.42). The factors associated with anxiety were moderate (PR: 1.92) and very/extreme worry (PR: 2.25) about working in a COVID-19 environment, moderate (PR: 1.26) and very/extreme (PR: 1.85) and worry about being marginalized by the neighborhood environment.

**Conclusions:**

There is an urgent need for action to address the mental health of these professionals, who have played a critical role in pandemic response and care.

## Introduction

Healthcare personnel are a critical group in healthcare due to the pandemic scenario resulting from exposure to SARS-CoV-2. This healthcare workforce (HW) led the front line of care from March-2020, therefore they have been continuously exposed to an increased risk of infection (1). and significant emotional burden. A high prevalence of depressive and anxious symptoms has been reported in this population (2–5), which has affected their ability to provide quality and effective patient care (6). The prevalence of depression has been reported as 65% in Chile (7), 62.2% in a multicenter study including Colombia, Ecuador, Bolivia, and Argentina (8), and 44.7% in Mexico (9), contrasting with lower rates in China (16.5%) (10) and the United States (38%) (11), similar to a meta-analysis showing 37% (12). For anxiety, prevalences were 74% in Chile (7), 40.1% in the same multicenter study (8), and 83.1% in Mexico (9), compared to 28.8% in China (10), 38% in the United States (11), and 40% in a meta-analysis (12).

In Peru, the Ministry of Health (MINSA) has issued guidelines emphasizing the protection of HW mental health (13). However, implementation has been uneven, particularly in peripheral regions like Lambayeque and Piura, which were among the hardest hit during the first wave (14). These regions face multiple structural challenges, including high levels of informal employment, socioeconomic inequality, and limited public healthcare infrastructure (15–18). According to national statistics, poverty and extreme poverty affect a significant proportion of households in these areas, with gaps in access to quality health services (19). These contextual vulnerabilities likely exacerbated the psychological toll on healthcare workers during the pandemic, especially in high-demand institutions such as the EsSalud public hospitals included in this study. Although national initiatives such as EsSalud’s mental health programs have emerged, they remain insufficient to meet the demands of frontline staff (20).

Previous studies have identified multiple risk factors for HW mental distress: direct contact with patients with respiratory diseases (7), working in public or state hospitals (8), to be a resident physician (9), to be women (8), living alone (21) having a poor perception of health (10). On the other hand, being a physician, unlike other health care personnel (21), use personal protective equipment (22), years of experience (23), coping strategies (24) and have a social support network (25) have been associated with a lower prevalence of depressive and anxious symptoms.

Although multiple factors associated with depression and anxiety in healthcare workers have been studied globally, some variables such as physical activity, income-related stress (9), role within hospital services (11), and dimensions of sleep quality have been less frequently addressed in Latin American studies, especially within the Peruvian context.

Second, few Latino studies have conducted evaluation during the first pandemic wave (7, 10, 22). Third, although several studies have used regression models to explore mental health outcomes in healthcare personnel, few have incorporated a broad set of psychosocial and occupational variables, such as stigma, perceived marginalization, and concern about exposure to COVID-19 in public hospital settings in Latin America, as we propose in this study (7, 9, 24). Fourth, few studies have evaluated health personnel under a multicenter approach of sites with broad relevance in COVID-19 care (22, 23). Fifth, although most studies in the literature employ validated instruments, a subset of prior research conducted in non-clinical populations or without formal psychometric validation in healthcare personnel or the local context may present limitations in reliability and contextual validity (7, 9, 26).

This research has relevant implications for health service management. First, depressive and anxious symptoms in HW may negatively affect their performance and quality of patient care (2, 6, 27–29). Second, identifying associated factors may help hospital managers implement preventive and early interventions, such as stress management training, psychological support, and protective measures. Third, it provides insights into the pandemic’s impact on HW in these hospitals, supporting the development of strategies for future crises. Fourth, the findings contribute to understanding the psychological and social mechanisms affecting HW mental health during COVID-19, informing more effective policies and programs.

For all of the above, this research aims to determine the prevalence and factors associated with depression and anxiety in the HW during the COVID-19 pandemic in three EsSalud hospitals in the northern regions of Piura and Lambayeque — areas severely impacted by the pandemic. This approach is consistent with recent WHO recommendations to strengthen occupational mental health strategies, particularly in resource-limited healthcare systems disproportionately affected by the pandemic (30).

## Methods

### Design and type of study

We conducted a multicenter cross-sectional study between July and August 2020 in three Social Security (EsSalud) hospitals located in the northern regions of Piura and Lambayeque, Peru—areas severely affected during the first wave of the COVID-19 pandemic.

The present analysis uses data from the baseline assessment of a longitudinal study. Although the parent study has a longitudinal design, the current manuscript analyzes only the first measurement, corresponding to a cross-sectional dataset collected between August and December 2020.

### Population, sample and sampling

The study population consisted of HWHW (doctors, nurses, obstetrician, nursing technicians, medical technologist, biologist, psychologist, among others) who were working in the Social Security Hospitals (EsSalud) of the Lambayeque region (Almanzor Aguinaga National Hospital and Hospital Luis Heysen Incháustegui) and Piura region (Hospital Regional Cayetano Heredia), 2020 during the first wave of the COVID-19 pandemic between the months of August-December 2020.

Eligible participants were health workers who had worked for at least one month during the COVID-19 emergency in 2020, either on the front line (COVID-19 wards, ICUs, emergency) or non-front line (general services). Inclusion required active employment at the time of data collection and voluntary participation with signed informed consent.

Exclusion criteria included: health workers on temporary medical leave or remote work due to high-risk conditions; those with incomplete or invalid responses in the PHQ-9 or GAD-7 questionnaires; and refusals to participate after initial contact. Participants were selected using systematic random sampling from the official list of health workers scheduled for mandatory COVID-19 screening.

The sample size was estimated to detect a minimum difference of 35% in the prevalence of depression and anxiety symptoms between exposed and unexposed groups, with 80% power and a 95% confidence level, using the reference prevalence observed in Lai et al. (2020) (27). Based on this, 62 participants per hospital were required. Considering a 10% non-response and 10% rejection rate, the total required sample was 152. During recruitment, 202 individuals were approached: 9 declined participation and data from another 11 were excluded due to incomplete or inconsistent responses (missing key variables). The final analytical sample comprised 182 participants.

A systematic random sampling was carried out, selecting the first participant randomly and the next with a fixed jump (every 10 participants). The list of health personnel in each hospital center was requested, differentiated by exposure groups (working and not working on the front line of COVID-19 health care). The choice of hospital centers was based on convenience, in the EsSalud Hospitals of Lambayeque and Piura. This research was based on primary data collected specifically for this study through direct field application of validated instruments, and does not correspond to a secondary data analysis.

### Variables

All variables were collected through structured face-to-face interviews conducted by trained personnel during the first wave of the COVID-19 pandemic (May to August 2020). The survey incorporated standardized psychometric instruments and a structured questionnaire designed to capture sociodemographic, occupational, physical health, and psychosocial characteristics.

#### Dependent variables

Two main outcomes were evaluated:

•Depressive symptoms, measured using the PHQ-9 scale, categorized as: minimal (0–4), mild (5–9), moderate (10–14), moderately severe (15–19), and severe (20–27).

•Anxiety symptoms, assessed using the GAD-7 scale, categorized as: mild (5–9), moderate (10–14), and severe (15–21).

Both outcomes were also analyzed as binary variables for sensitivity models: PHQ-9 ≥10 and GAD-7 ≥10, indicating clinically relevant symptoms.

#### Independent variables

1. Personal variables: age in years, sex (male, female), marital status (single, married, cohabiting, widowed), religion (none, Catholic, non-Catholic), having children (no, yes), role in the father/mother family (no, yes).
2. Physical health variables: comorbidities (high blood pressure, diabetes mellitus, obesity), diagnosis of COVID-19 (no, yes)
3. Labor variables: monthly family income based on the amount of Minimum Living Wage (SMV) of the Peruvian state as of 2020 (1 to 3, 3 to 5, 5 to 7, 7 to 10, more than 10), type of profession (doctors, nurses, obstetrician, nursing technicians, medical technologist, biologist, psychologist, dentist, others), role in the service where they work (leader, leader support, operational staff), and type of contract (appointed, Administrative Service Contracts- CAS, third-party service, resident, other).
4. Psychosocial variables: resilience (low, high), psychological, physical or sexual abuse (no, yes), compliance with confinement measures (no, yes) and social distancing (no, yes), perception of the pandemic (very serious, serious, mild, not serious), personal history of mental health problems, defined as a self-reported positive answer to the question: “Have you ever been diagnosed or treated for a mental health problem before the COVID-19 pandemic?” (no, yes), trust in the government to handle the pandemic (no, yes), history of a close family member recently infected with COVID-19 (no, yes), history of a close family member recently dying from COVID-19 (no, yes), worry about getting COVID-19 (not at all, a little, moderately, a lot, extremely), worry about infecting family members with COVID-19 (not at all, a little, moderate, a lot, extremely), concern about availability of Personal Protective Equipment (PPE) (not at all, a little, moderate, a lot, extremely), concern about not having health insurance (not at all, a little, moderate, a lot, extremely), concern about working in COVID-19 environments (not at all, a little, moderate, a lot, extremely), concern about being marginalized due to having been exposed to SARS-CoV-2 infection (not at all, a little, moderate, a lot, extremely), concern about being an asymptomatic patient (not at all, a little, moderate, a lot, extremely), insomnia (no, subclinical, clinical moderate, clinical severe), level of physical activity (low, moderate, high), burnout syndrome (no, yes), eating disorder (no, yes), tobacco consumption (low, moderate, high) and alcohol consumption (low, moderate, high).

### Procedures

A structured and systematic recruitment process was employed to minimize selection bias. Healthcare workers (HW) selected through systematic random sampling from institutional screening lists were invited to participate. Those who agreed provided electronic informed consent after receiving detailed information about the study. Although participation was voluntary, the recruitment followed a closed and sequential process based on predefined sampling frames.

Data were collected through in-person interviews using structured questionnaires administered by trained field personnel. Interviewers—health professionals trained by the research team—applied the instruments directly at each healthcare worker’s workplace (HWHW) within EsSalud facilities. The questionnaire included sociodemographic, psychosocial, occupational, and physical health variables.

Mental health outcomes were evaluated using a specific temporal framing: “Have you ever experienced any of the following symptoms (…) due to your care work since the beginning of the COVID-19 health emergency?”

REDCap^®^ software was used to design the questionnaires, collect data digitally, and implement real-time quality control. Interviews were conducted in designated care modules that ensured biosafety standards, including adequate ventilation, physical distancing, and the use of personal protective equipment. Each interview lasted approximately 20 minutes.

### Instruments

#### Generalized Anxiety Disorder 7-Item Scale

The GAD-7 consists of 7 questions and evaluates the frequency of symptoms during the last 2 weeks (31). It was validated among a group of immigrants of Hispanic origin living in the United States (32). The cut-off points used were 0 to 4 as normal, 5 to 9 as mild, 10 to 14 as moderate, and 15 to 21 as severe. The cut-off point of 5 or more has been validated, finding optimal psychometric properties with a positive predictive value and negative predictive value greater than 0.80 (33). It has been validated in health personnel in Latin America during the COVID-19 pandemic, and specificity of 0.83 and sensitivity of 0.92 have been estimated. In addition, it has been used in health personnel during the COVID-19 pandemic (33).

#### Patient Health Questionnaire-9

The PHQ-9 is a self-administered version measured according to a Likert scale that consists of nine criteria to evaluate major depression (34)., among the categories, 0 to 4 are rated as normal, 5 to 9 as mild, 10 to 14 as moderate, and 15 to 21 as severe. With a cut-off point greater than and equal to 5, it represents an optimal compromise between sensitivity with 0.92 and specificity of 0.89 (35). It has been solidly validated in a representative population of Peru, through research obtained from data from the Encuesta Demográfica y de Salud Familiar (ENDES), in which optimal invariance and reliability were estimated (36). It has been validated in patients using primary health care in Latin America during a pre-pandemic scenario due to COVID-19 with a Cronbach’s alpha of 0.89 (37).

A cut-off score of ≥5 was used for PHQ-9 and GAD-7 to capture the presence of at least mild symptoms, which is supported by prior literature in high-risk healthcare populations. This threshold was chosen to improve sensitivity for early detection and public health response, recognizing that it does not imply clinical diagnosis (38–43).

#### Connor-Davidson Abbreviated Scale

The CD-RISC consists of 10 items that measure resilience globally through a Likert scale with a score of 0–4, whose relationship between the score and presence of resilience is directly proportional. and is classified according to quartiles (low: first quartile, moderate: second and third quartile, high: fourth quartile) (44). It has been validated in older adults (45), health personnel (46), young adults (47) and Spanish-speaking fibromyalgia patients (48). It has been used in the COVID-19 context in university students (49).

#### Insomnia Questionnaire

The ISI consists of seven items that measure self-reported perception of the severity of insomnia through a 0–4 point Likert scale whose final score varies between 0–28 points (50). The score is categorized as follows: normal from 0 to 7, subthreshold or mild from 8 to 14, moderate to severe from 15 to 21 and severe from 22 to 28. It has been validated in older adults (51), primary care patients (52) and general Spanish-speaking population (53). It has been used in the COVID-19 context in the general population of a Latin American country (54).

#### Eating Disorder Questionnaire

The EAT-26 consists of 26 questions measured using a Likert scale with six response options (“never”, “rarely”, “sometimes”, “a often”, “very often” and “always”, using 20 as a cut-off point to assume an eating disorder. The cut-off value of >= 11 has been validated in a study in the female population (55); and male in Colombia (best cut-off value: ≥20 points) (56). It has been used in the COVID-19 context in a multicenter study of a university population in a Latin American country (57).

#### Substance Use Questionnaire

ASSIST consists of 8 questions that inquire about substance use in the last 3 months. It has been evaluated by the World Health Organization (WHO) and has been validated in the Spanish-speaking population, with adequate internal consistency globally and specifically according to the type of substance (58). With a reliability of Cronbach’s Alpha = 0.84) (59). The type of risk that exists for tobacco and alcohol consumption is interpreted: low (0 to 10 points), moderate (11 to 26 points) and high (27 to maximum) (60). It has been used in the COVID-19 context in a population at risk in a Latin American country (59).

#### Physical Activity Questionnaire

The IPAQ-S consists of 9 items and evaluates physical activity reported in the last 7 days (61). Allows you to obtain a weighted estimate of total physical activity from the activities reported per week, to categorize physical activity as: Intense, moderate, light or inactive (61). It has been validated in Spanish-speaking populations and applied in the Latin American population (61–63). With a reliability level of 0.80 (64). The short version has shown significant correlations (0.26-0.69) in relation to accelerometer measurements in Spanish-speaking populations (65). It has been used in the COVID-19 context in the university population (66).

#### Maslach Burnout Inventory Human Services Survey

Considered the gold standard for measuring Burnout Syndrome, the MBI-HSS consists of 22 items and 3 domains: Emotional exhaustion, depersonalization and personal fulfillment at work; and has been validated in Chilean and Peruvian health workers (67, 68). With a high internal consistency > 0.70 (69). For its identification in Peruvian health personnel, it is recommended to use cut-off points predetermined by the creator of the instrument (AE > 26, DP >9 RP < 34) (68). It has been used in a post-pandemic context in frontline defense personnel (70).

The internal consistency of all psychometric instruments was assessed within our study sample. Reliability was high for the GAD-7 (Cronbach’s α = 0.88), PHQ-9 (α = 0.82), CD-RISC-10 (α = 0.91), Insomnia Severity Index (α = 0.85), and EAT-26 (α = 0.88). The alcohol and tobacco subscales of the ASSIST showed acceptable internal consistency (α = 0.68), and the IPAQ activity items demonstrated expected reliability for multidomain behavioral measures (α = 0.65).

### Data analysis plan

All statistical analyses were performed using STATA v18^®^. The primary objective was to examine whether depressive and anxiety symptoms were associated with sociodemographic, occupational, psychosocial, and health-related factors among HWs.

Missing data was handled through a complete-case approach, as the dataset was nearly complete for all primary variables. No imputation procedures were performed because the outcome variables were 100% complete. Only minimal missingness was observed in secondary covariates. Given the low proportion and random distribution of missing data, listwise deletion was applied without compromising statistical validity.

### Descriptive analysis

Univariate statistics were used to describe the sample. Categorical variables were summarized using absolute and relative frequencies (%). For continuous variables, means and standard deviations (SD) were reported for normally distributed data, while medians and interquartile ranges (IQR) were presented for non-normally distributed variables.

### Bivariate analysis

To explore crude associations between each outcome (depression or anxiety) and independent variables: Chi-square or Fisher’s exact tests were applied for categorical comparisons. Mann–Whitney U tests were used for continuous variables due to non-normal distribution. This exploratory step allowed the identification of candidate variables for multivariate modeling, based on a significance threshold of p < 0.05.

### Multivariate analysis

To estimate adjusted associations, we fitted Generalized Linear Models (GLM) of the Poisson family with a log link and robust standard errors. Hospital site was included as a clustering variable to adjust for intragroup correlation.

Variables were selected based on both conceptual and statistical criteria. Theoretical and empirical evidence from previous studies guided the inclusion of factors with established or plausible associations with depression and anxiety among healthcare workers during the COVID-19 pandemic. From the statistical standpoint, variables showing a p-value < 0.05 in simple Poisson regression models were entered into the multivariable model.

#### Model 1

Included covariates with p < 0.05 in univariate Poisson regressions, based on outcomes defined using low-threshold cutoffs (PHQ-9 ≥5 and GAD-7 ≥5), to capture early symptoms relevant for occupational health surveillance.

#### Model 2

Used higher cutoffs (PHQ-9 ≥10 and GAD-7 ≥10) to define clinically significant depressive or anxiety symptoms. This model followed the same inclusion rules as Model 1.

Collinearity diagnostics using Variance Inflation Factor (VIF) were applied to all final models (VIF < 2.5), confirming no relevant multicollinearity.

### Sensitivity and confounder adjustment (Model 3)

A third model was constructed to assess the robustness of associations by adjusting for theoretically relevant confounders.

Model 3 included all variables statistically significant in Model 2, plus key covariates identified in the literature as potential confounders—regardless of their bivariate significance—including: age, sex, resilience, insomnia, physical activity, and tobacco use. Model 3 was estimated with the same GLM-Poisson approach and hospital-level clustering.

To visually summarize the associations between the covariates and the outcomes (depressive and anxiety symptoms), forest plots were generated from the multivariable Poisson regression model 1. All forest plots were produced using the R statistical.

## Results

The majority were women (62.6%) and the median age was 38.9 years. 33.9% reported having had COVID-19, 12.1% were obese, and 5.5% reported having high blood pressure. Regarding the occupational group, 48.4% were doctors and 22% were nurses. 46.7% reported experiencing mistreatment while providing health care to COVID patients. 77.5%, 60.4%, 52.8%, 40.1% and 39% reported having very/extreme concern about infecting their family members with COVID-19, becoming infected with COVID-19, availability of PPE, working in a COVID environment and being an asymptomatic patient; respectively. 23.6% had a low level of resilience, 21% had subclinical insomnia and 42.9% had a high level of physical activity. 6.1% and 7.1% presented an eating disorder and Burnout Syndrome; respectively. 42.9% presented depressive symptoms (95% CI: 35.56-50.39). Among those with depressive symptoms (42.9%), 31.9% had mild symptoms and 9.3% had moderate symptoms. Regarding anxiety, 50.6% of the total participants (95% CI: 43.05–58.03) reported anxiety symptoms. The majority presented mild anxious symptoms (37.4%), however, 6.0% and 7.1% presented moderate and severe anxious symptoms, respectively (**Supplementary Table 1**).

**Supplementary Table 2** shows the characteristics associated with depressive and anxious symptoms in health personnel. In relation to depressive symptoms, it was found that women presented a higher frequency of depressive symptoms, compared to men (49.1% vs. 32.4%; p=0.027). A higher frequency of depressive symptoms was found in health personnel with a history of COVID-19 (56.1% vs. 37.8%; p=0.024), compared to those who had not yet been infected. A higher frequency of depressive symptoms was observed in health personnel with a family member infected with COVID-19, compared to those who did not have such a history (50% vs. 32.9%; p=0.021). Health personnel who reported being very/extremely worried about infecting their family members with COVID-19 (46.8%) and being asymptomatic patients (56.3%) had a higher frequency of depressive symptoms. Health personnel with insomnia had a 38.9% higher frequency of depressive symptoms, compared to those who did not have insomnia (73.2% vs. 34.3%; p<0.001). Health personnel with a moderate level of physical activity had a higher frequency of depressive symptoms; compared to those who had a low level of physical activity (70.6% vs. 37.9%; p=0.045).

**Figure 1:**
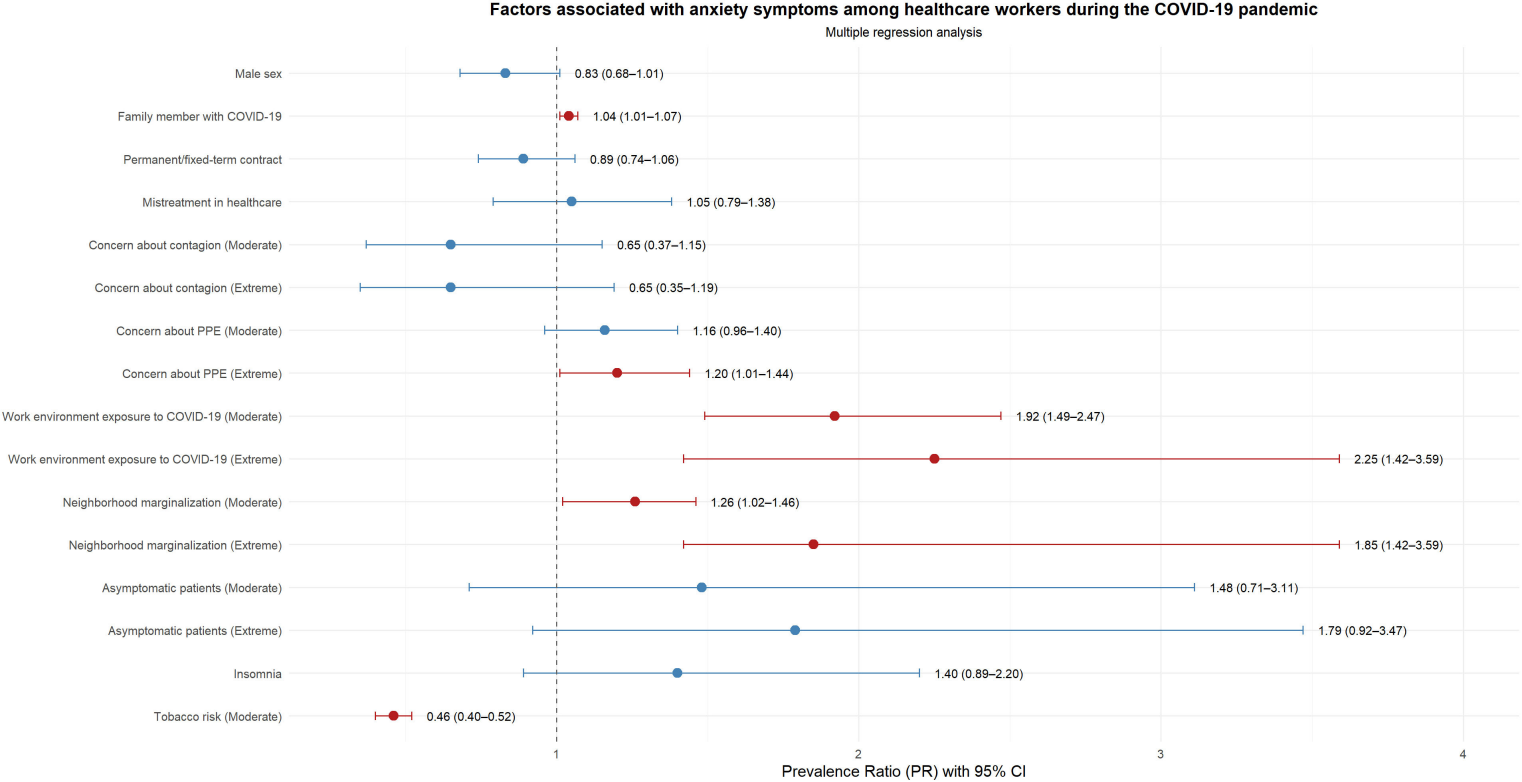
Forest plot of factors associated with anxiety symptoms among healthcare workers during the COVID-19 pandemic (Model 1).

Regarding anxious symptoms, the highest frequency of anxious symptoms was found in women (57% vs. 39.7%; p=0.024). Health personnel who reported not complying with social distancing measures presented a higher frequency of anxious symptoms, compared to those who did comply with these measures (76.2% vs. 47.2%; p=0.012). Having experienced some type of abuse during care implied a greater frequency of anxious symptoms; compared to those who did not receive said abuse (58.8% vs. 43.3%; p=0.037). Additionally, a higher frequency of anxious symptoms was identified in health personnel who reported being very/extremely worried about infecting their family members (58.2%), availability of PPE (57.3%), working in a COVID environment (65.8%), being marginalized by neighborhood environment (88.9%) and being an asymptomatic patient (71.8%); These variables were statistically significant. Health personnel with insomnia presented a 19.5% higher frequency of anxious symptoms, compared to those who did not have insomnia (65.9% vs. 46.4%; p=0.029) (**Supplementary Table 2**, **Figure 1**).

**Figure 2:**
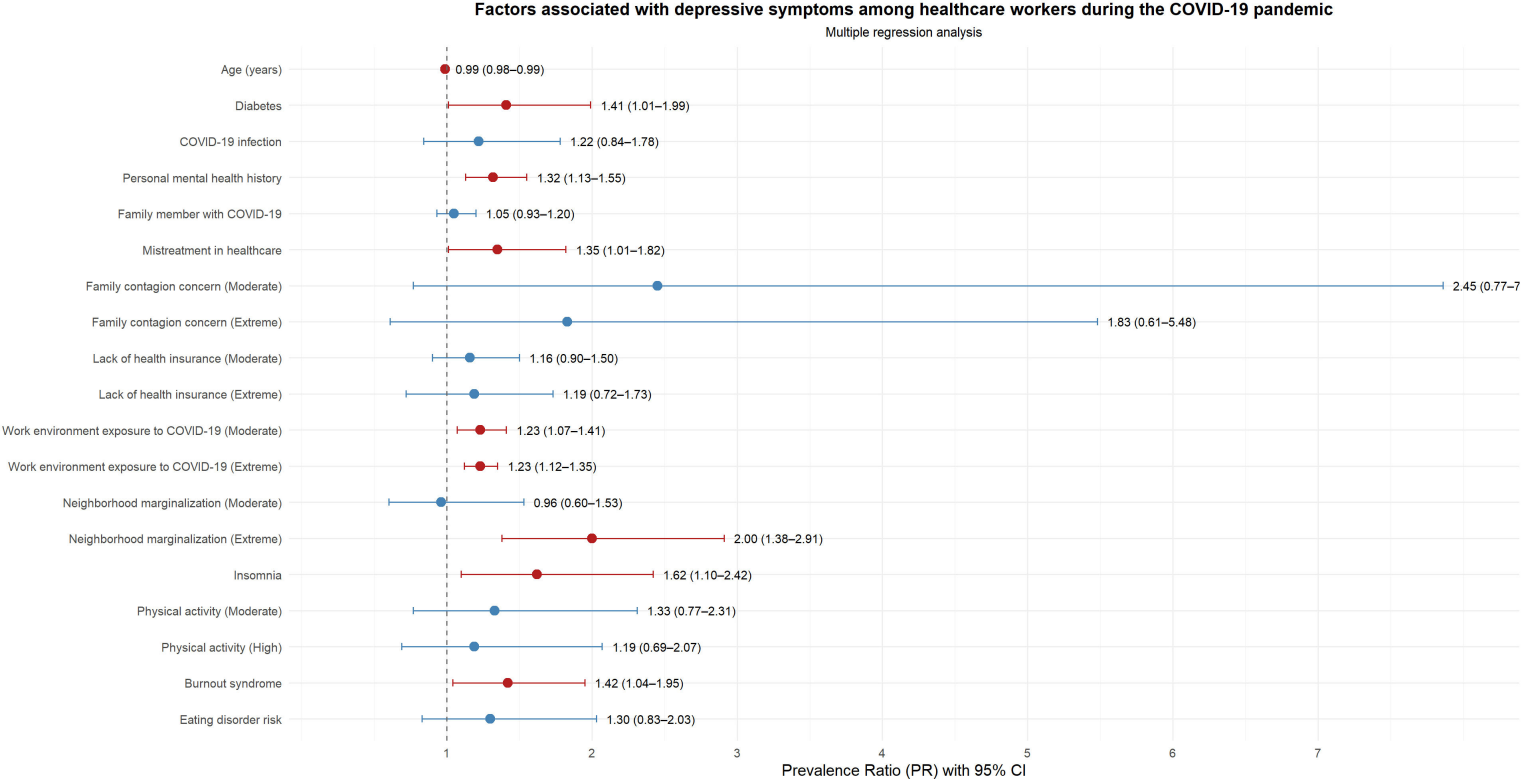
Forest plot of factors associated with depression symptoms among healthcare workers during the COVID-19 pandemic (Model 1).

In the multiple regression analysis, a higher prevalence of depressive symptoms was associated with being diabetic health personnel (PR: 1.41; 95% CI: 1.01-1.99), having a previous history of mental health (PR: 1.32; 95% CI: 1.13 -1.55), having experienced abuse during health care (PR: 1.35; 95% CI: 1.01-1.82), having moderate concern about working in a COVID environment (PR: 1.23; 95% CI: 1.07-1.41), having a lot/extreme concern about working in a COVID environment (PR: 1.23; 95% CI: 1.12-1.35) and having a lot/extreme concern about being marginalized due to the neighborhood environment (PR: 2.00; 95% CI: 1.38-2.91). Insomnia was associated with a 62% higher prevalence of depressive symptoms (PR: 1.62; 95% CI: 1.10-2.42). Burnout syndrome was associated with a 42% higher prevalence (PR: 1.42; 95% CI: 1.04-1.95). Each additional year of age was associated with a 1% lower prevalence of depressive symptoms (PR: 0.99; 95% CI: 0.98-0.99) (**Supplementary Table 3**, **Figure 2**).

In the sensitivity analysis using the PHQ-9 ≥10 cut-off (Model 2), several associations observed in the main model were no longer statistically significant—namely age, diabetes, insomnia, and concern about working in a COVID-19 environment. However, key associations remained consistent, including mistreatment in healthcare settings, burnout syndrome, and high levels of perceived neighborhood marginalization. Notably, the magnitude of the association between burnout syndrome and depressive symptoms increased substantially in Model 2 (PR: 5.73; 95% CI: 1.77–18.48), underscoring its central role in clinically significant depressive symptoms. The complete results are available in **Supplementary Table 4**.

The sensitivity analysis using a GAD-7 ≥10 cut-off (Model 2) revealed notable changes in the pattern of associated factors. Several associations observed in Model 1 were no longer significant in Model 2—specifically, having a family member with COVID-19, concern about personal protective equipment availability, neighborhood marginalization, and tobacco use. However, concern about working in a COVID-19 environment remained significantly associated with a higher prevalence of anxiety in both models, showing a substantial increase in the strength of the association at the higher cut-off (PR: 7.63; 95% CI: 1.15–50.69 in Model 2 vs. PR: 2.25 in Model 1). New associations emerged in Model 2 as well, including being a physician (PR: 0.56; 95% CI: 0.42–0.74), insomnia (PR: 2.53; 95% CI: 1.52–4.22), eating behavior disorder (PR: 2.30), and burnout syndrome (PR: 1.40). These results are summarized in **Supplementary Table 4**.

In relation to anxious symptoms in health personnel, having a lot/extreme concern about the availability of PPE was associated with a 20% higher prevalence (PR: 1.20; 95% CI: 1.01-1.44). Moderate and high/extreme concern about working in a COVID-19 environment were associated with a 92% (PR: 1.92; 95% CI: 1.49-2.47) and 125% (PR: 2.25; 95% CI: 1.42- 3.59) higher prevalence of anxiety symptoms. Moderate and high/extreme concern about neighborhood-related marginalization were associated with 26% (PR: 1.26; 95% CI: 1.02-1.46) and 85% higher prevalence (PR: 1.85; 95% CI: 1.42-3.59); respectively. Moderate tobacco consumption was associated with a lower prevalence of anxiety symptoms (PR: 0.46; 95% CI: 0.40-0.52) (**Supplementary Table 3**).

In Model 3, which adjusted for both statistically significant and theoretically grounded variables, the main associations identified in Model 2 remained consistent in direction and magnitude. The inclusion of potential confounders did not materially alter the strength of most associations, supporting the robustness of our findings. Full results of this adjusted model are presented in **Supplementary Table 4**.

## Discussion

### Main findings and contextualization

This study adds novel insights to the growing literature on HW mental health during COVID-19, particularly within Latin America. Unlike prior studies that often relied on convenience samples or descriptive analyses (71), our research employed institution-based probabilistic sampling, robust multivariable models, and sensitivity analyses using different clinical thresholds. It is, to our knowledge, the first multicenter study conducted in northern Peru during the early pandemic wave, and one of the few to integrate underexplored psychosocial dimensions such as perceived neighborhood stigma, mistreatment in care, and fear of being asymptomatically infectious. These contextual variables strengthen the explanatory power of our models and highlight structural vulnerabilities in overstretched public healthcare systems.

### Prevalence of depression and anxiety symptoms

In our study, 42.9% of HW exhibited depressive symptoms—31.9% with mild and 9.3% with moderate levels. These figures are consistent with studies in Peru [Arequipa: 30.2% (2), Lima: 53.3–46.2% (3)] and Colombia [40.8% (23)], but lower than findings from multicountry Latin American studies [62.2% (8)] and Chile [65% (7)]. By contrast, lower rates were reported in China [34.3% (72)] and the U.S. [38% (11)], aligning with meta-analyses estimating pooled prevalence around 23 (73)–37% (74).

Regarding anxiety, 50.6% of participants showed symptoms (37.4% mild, 6.0% moderate, 7.1% severe), comparable to multicountry Latin American data [40.1% (8)], but higher than pooled estimates from global meta-analyses [22.8 (5)–29.6% (73)] and studies in China [18.1% (72)] or Arequipa, Peru [32.1% (2)]. Chile reported the highest prevalence [74% (7)]. Finally, to a meta-analysis that included health personnel who cared for patients with COVID-19 in whom I identified that the cumulative prevalence of anxiety was 25.8% (74).

The high frequency of symptoms of anxiety and depression could be explained by the sudden change from traditional work to the confinement of working in silence and without contact with peers, the constant fear of infection, poor social support, residing in regions with high rates. of exposure and infection by COVID-19 as was the Latin American continent, experiencing financial insecurities (75, 76). However, this difference with the continents of Europe and Asia could be attributed to the fact that LATAM presents inadequacies in health systems, absences in prevention or anticipation programs for the promotion of good mental health, and the cultural stigma associated (77) and socioeconomic inequalities, generating negative repercussions on people’s quality of life (78).

We adopted a PHQ-9 and GAD-7 cut-off of ≥5 based on both clinical relevance and managerial implications. Clinically, this threshold captures early or subthreshold symptoms, which may not yet meet diagnostic criteria but still impair emotional well-being and work performance (79, 80). From a health services perspective, this lower threshold enables broader mental health surveillance, guiding timely support and preventing deterioration. It also generates actionable data for human resources planning, including the design of workplace interventions and psychological support programs. Importantly, we do not interpret this cut-off as a diagnostic label but as a pragmatic tool for early detection during public health emergencies.

### Factors associated with symptoms of depression

Each additional year of age was associated with a slightly lower prevalence of depressive symptoms (approximately 1%). This finding aligns with studies reporting lower depression rates in older adults, possibly due to greater emotional regulation or resilience developed with age (81–83). However, other studies have reported either a direct age-depression relationship (84) or higher risk of depressive symptoms among adolescents and young adults (85). Although the biological mechanism remains unclear, resilience and accumulated experience in managing stress may explain this inverse association in healthcare settings (86–88).

Having diabetes as a comorbidity was associated with a 41% higher prevalence of depressive symptoms. This is consistent with studies linking chronic comorbidities with increased emotional distress in healthcare personnel (89, 90). Likewise, Lucas-Hernandez et al., in Mexico during the pandemic identified a greater frequency of depressive symptoms in people with diabetes (9). The psychological burden of managing a chronic illness, combined with pandemic-related stress, may contribute to this association (91–93).

Having a previous personal history of mental illness was associated with a 32% higher prevalence of depressive symptoms. This is similar to what was reported by Valencia A. et al., who among Colombian health personnel during the pandemic identified that those with previous depression were more likely to present depression again (OR = 2.5) (23). This association could be due to the fact that health personnel, such as those who cared for patients with COVID-19, are more emotionally vulnerable and are more likely to seek mental support for their problems; it may also be explained by a lack of access to mental health services (94).

Having experienced abuse during health care was associated with a 35% higher prevalence of depressive symptoms. This finding is consistent with what was described by Want et al. who in their study carried out on health workers from 31 provinces in China found that violence reported in the work environment was more likely to have mental health symptoms during the COVID-19 pandemic (b=8.248) (95). Likewise, it agrees with what was reported in systematic reviews which suggest a significant increase in violence in health workers during the pandemic (96–98). Mistreatment may exacerbate stress, lower self-esteem, and contribute to feelings of hopelessness, thereby heightening the risk of depression (97, 99).

Moderate to extreme concern about working in a COVID-19 environment was associated with a 23% higher prevalence of depressive symptoms, in line with findings from southern Peru (100). Moderate or extreme concern about the work environment can expose healthcare personnel to a greater burden of stress and anxiety, as they fear facing challenging or risky situations in their work environment (101). Uncertainty about job security (102), Fear of exposure to infectious diseases, scarcity of resources, and emotional exhaustion are factors that could contribute to the appearance of depressive symptoms. Furthermore, constant worry can have a negative impact on the psychological and physical well-being of healthcare workers, which in turn increases the likelihood of experiencing depressive symptoms (103–105). These stressors highlight the need for psychosocial support interventions.

Extreme concern about neighborhood stigma related to COVID-19 exposure was associated with a two-fold higher prevalence of depressive symptoms. This is supported by studies from Japan (106) and Colombia (23, 107) showing that perceived discrimination strongly correlates with depression among healthcare workers. This association could be explained by the fact that exposure to a pandemic such as COVID-19 can generate stigmatization and discrimination towards those health workers who are in the first line of defense against the disease (108). Concern about being marginalized or rejected by their neighborhood environment due to their exposure can generate a constant state of alert and anxiety in health personnel (109). Additionally, uncertainty about the future and the possibility of being stigmatized can lead to excessive worry and increased anxious reactivity (110). The chronic stress associated with this worry can also have a negative impact on the psychological and physical well-being of health personnel, contributing to the development of anxious symptoms (110).

Having insomnia was associated with a 62% higher prevalence of depressive symptoms. This is consistent with that of Zhang et al, who in research conducted in China found that depression and insomnia were significantly correlated, after controlling for the effects of sex, age, education, smoking and alcoholism in medical personnel. front line of care against COVID-19 (111). Likewise, Valencia A. et al., in Colombian health personnel identified that those with previous insomnia were more likely to present depression (OR = 2.0) (23). This bidirectional relationship may be exacerbated in high-stress occupational settings, such as during the COVID-19 pandemic, where increased workloads, shift work, and concerns about safety may disrupt sleep patterns. Insomnia not only compromises emotional regulation but also reduces resilience to stress, potentially increasing vulnerability to mental health disorders. These findings highlight the need for institutional support strategies to address sleep hygiene and mental well-being in healthcare settings.

Health personnel with burnout syndrome were associated with a 42% higher prevalence of depressive symptoms. This is contrary to what was reported by García-Torres et al., who in their study of Mexican health personnel identified a correlation between SB and depressive symptoms (r= 0.217) (112). Similarly, Babamiri et al., in their study conducted on Iranian health personnel, identified that emotional exhaustion (EB) increased the probability of having negative mental health symptoms (OR = 6.92) (113). Likewise, Karadag et al., in their study conducted in Turkey during the pandemic in health personnel in the intensive care unit, identified a moderate and significant correlation between SB and depression symptoms (r = 0.519) (114). It is highlighted that there is little research on these variables in health personnel, therefore, with the present result it adds to the current literature. The high prevalence could be due to the fact that being a health personnel means exposing oneself to the main factors that contribute to the development of BS, such as the stress generated by hospital conditions with excessive workload, exposure to suffering and death of patients (115). Emotional and physical demands, lack of equipment and materials, long hours and days, among others (116). However, the biological mechanism between both variables has not been identified.

### Factors associated with anxiety symptoms

Health personnel who reported having little fear of contracting COVID-19 were associated with a lower prevalence of anxiety symptoms in the simple model, however: this was diluted in the multiple model. Similar patterns have been reported in Iran (117), Jordan (118), and Turkey (119), where fear of infection correlated positively with anxiety symptoms among healthcare staff. The lower prevalence of anxious symptoms among those with little fear of becoming infected can be explained by several psychological and emotional factors (120). Excessive or chronic fear can generate high levels of anxiety and worry in healthcare workers, negatively affecting their emotional and physical well-being (121). Conversely, those with less fear may have a more realistic and balanced perception of the risks associated with exposure to the virus, which could help reduce anxiety (122). Additionally, healthcare personnel who feel less fear may have a greater sense of control and security in their work environment, which may contribute to a lower likelihood of developing anxious symptoms. It is also possible that those with less fear are better equipped to handle the stress and challenging situations associated with the pandemic (123).

Concern about inadequate access to PPE was associated with higher anxiety prevalence, consistent with studies from Colombia (23, 124) and Peru (22), where lack of PPE increased risk of mental health symptoms among health personnel. These results are related to what we know about prevention measures to reduce the risk of becoming infected by SARS-CoV-2, which would make health personnel calmer and more confident if they work with PPE. It has been shown that Peru has had weaknesses in its response to the pandemic due to the fact that in recent years it has experienced a political crisis and a fragmented health system (22, 125). Situation that had greater risk in state establishments, with which health personnel could significantly experience the development of these negative psychiatric symptoms (126).

Perceived marginalization by neighbors due to COVID-19 exposure was associated with a higher prevalence of anxiety symptoms, echoing findings from Jordan (127) and Iran (128). However, it differs from what was reported in Nepal, in which no association was found between anxiety and stigma in health personnel (129). Health personnel who have been marginalized by people in their neighborhood due to their exposure to COVID-19 infection may experience feelings of isolation, rejection, and stigmatization (130). This perception of being excluded or judged for their work in addressing the pandemic can generate additional emotional burden and increase anxiety levels in these workers (131). Additionally, a lack of support and understanding from the community can lead to fears about their own safety and that of their loved ones, contributing to anxiety (132). Stigmatization and marginalization can have a negative impact on the emotional and psychological well-being of health personnel, affecting their self-esteem and self-confidence (133). These factors may predispose them to developing anxious symptoms and affect their ability to cope with stress related to their work in COVID-19 care (133).

Having moderate concern about working in a COVID-19 environment was associated with a 92% higher prevalence of anxiety symptoms, while having great/extreme concern was associated with a 125% higher prevalence. This is similar to what was reported in Peruvian studies, which have identified that fear of COVID-19 is positively associated with the development of anxious symptoms in the general population (134). and health workers (36). This association could be explained because the work environment in COVID-19 care presents unique and stressful challenges for health personnel (135). Continued exposure to the virus, uncertainty about your own health and that of your loved ones, the need to follow strict safety protocols, and the emotional burden of treating patients with a life-threatening illness can lead to high levels of anxiety and worry in patients. healthcare workers (120). Additionally, the constant concern about the possibility of contagion and the responsibility of providing quality care in the midst of the pandemic can lead to a state of permanent alert and tension. Furthermore, the fear of becoming infected and the perception of being at constant risk can affect the emotional and physical well-being of health personnel, predisposing them to develop anxious symptoms (120).

Moderate tobacco consumption was associated with a 54% lower prevalence of anxiety symptoms. This differs from those reported by Lucas-Hernández et al., who in their study carried out on Mexican health personnel during the pandemic identified a greater frequency of depressive symptoms in those who used tobacco (70% vs. 30%) (9). Contrary to what was reported by Stanton et al., who in Australian adults during the pandemic identified that the negative change in tobacco consumption was associated with the greater development of anxiety symptoms (OR = 1.12) (136). Finally, Bassi et al., in their study in the general population of India during the pandemic, identified that tobacco users presented more anxiety symptoms by 20.7% more (137).

Having moderate worry about being an asymptomatic COVID-19 patient was associated with a 49% higher prevalence of anxiety symptoms, and having very/extreme worry was associated with an 80% higher prevalence. However, in the multiple model the association was diluted. Although no previous studies have been identified that have exactly analyzed this association, there is solid evidence that has identified an association between fear of COVID-19 and anxiety (117, 119, 120). This association could be due to the fact that the concern about being asymptomatic implies a constant fear of carrying and transmitting the virus without presenting visible symptoms, which generates a feeling of uncertainty and lack of control over one’s own health and the possibility of infecting others (138–140). Persistent worry about this possibility can lead to continued alertness and anxiety (138–140). Furthermore, health personnel are on the front line of combating the disease, constantly facing the virus in their work, which can intensify their worry and stress. The fear of becoming infected and the responsibility of staying healthy to continue providing care to patients can put additional pressure on these workers (138–140).

### Sensitivity analysis for depressive and anxiety symptoms using PHQ-9 and GAD-7 ≥10 cut-off

The comparison between both models reinforces the validity of core risk factors and highlights how the choice of psychometric thresholds can shape epidemiological patterns. While a PHQ-9 cut-off of ≥5 is useful for identifying early or mild symptoms in occupational screening, using ≥10 captures more severe cases, often requiring clinical attention. The observed loss of significance in some demographic and clinical variables under the ≥10 cut-off may reflect their influence on subclinical or transitional symptom states. This dual analytical approach provides a more comprehensive understanding of the depressive symptom spectrum among healthcare workers and strengthens the public health and occupational implications of our findings. This sensitivity analysis reinforces the central role of certain risk factors in clinically significant anxiety and illustrates the influence of the chosen threshold on the interpretation of epidemiological data. While a GAD-7 cut-off of ≥5 allows for early detection of mild or subthreshold anxiety symptoms—important for occupational health surveillance—the ≥10 threshold highlights more severe clinical manifestations. The shift in significance and emergence of new associations under the higher threshold suggests that some psychosocial and clinical variables are more predictive of moderate-to-severe anxiety. The use of both cut-offs provides a broader epidemiological perspective, integrating early detection with clinically meaningful outcomes, and strengthening the practical implications for mental health support among healthcare workers.

Additionally, the inclusion of a third model adjusting for theoretically plausible confounders (e.g., age, sex, resilience, insomnia, physical activity, and tobacco use) further enhanced the robustness of our findings. This model confirmed the stability of key associations while accounting for residual confounding, offering a more rigorous assessment of the factors linked to mental health outcomes in healthcare workers.

### Implication of online findings from health services management research in the Peruvian context

Our findings provide timely evidence to inform occupational health policies within the Peruvian public health system, one of the most severely impacted in Latin America during the COVID-19 pandemic (141). The high prevalence of depressive and anxiety symptoms observed among public-sector healthcare workers especially those experiencing workplace mistreatment, high perceived marginalization, or burnout highlights the urgent need for institutional responses tailored to this population’s needs.

Based on our results, we recommend implementing structured mental health screening programs within hospitals, prioritizing frontline staff and those with known occupational vulnerabilities (e.g., limited job security, high workload, or comorbidities). Interventions should include regular psychological evaluations, on-site mental health services, and referral pathways for specialized care. In addition, promoting protective factors such as physical activity, organizational justice, and healthy workplace environments should be integrated into occupational health protocols.

These measures must be aligned with existing national mental health frameworks (e.g., the *Plan Nacional de Fortalecimiento de Salud Mental*), but operationalized at the institutional level to ensure coverage, continuity, and cultural relevance. Preparing contingency mental health support systems for future health emergencies is also essential, especially in under-resourced public facilities. Ultimately, ensuring the mental well-being of healthcare workers is not only an ethical imperative, but also a critical component of health system resilience in Peru and similar middle-income countries.

Our findings support WHO’s updated recommendations on protecting the mental health of healthcare workers during and beyond the COVID-19 pandemic, particularly in vulnerable systems such as Peru’s public health sector (142).

### Limitations and strengths

This study presents several limitations that should be considered when interpreting the results. First, due to its cross-sectional design, causal inferences cannot be drawn. The identified associations are exploratory and should be interpreted as correlational, even though we adjusted for potential confounders using multivariable regression. The study serves to generate hypotheses for future longitudinal research.

Second, self-administered questionnaires may have introduced measurement bias. Additionally, unmeasured confounding variables—such as postgraduate education, housing-related financial stress stress (137), or specific work units (e.g., ICU, emergency) (143) could have influenced the outcomes, particularly in the context of depression and anxiety.

Third, although systematic random sampling was used from COVID-19 screening rosters, only individuals present and consenting at the time of data collection were included. This may have excluded those absent due to illness, psychological distress, or other factors, introducing potential selection bias. As a result, prevalence estimates might be over- or underestimated.

Fourth, although our models incorporated hospital-level clustering using robust standard errors, we did not account for clustering at the unit or professional level. This may have led to underestimated standard errors and overestimated precision in some associations. Future studies should consider hierarchical or multilevel modeling to better reflect the nested structure of institutional settings.

Fifth, the absence of pre-pandemic mental health data prevents direct attribution of findings to the COVID-19 emergency. However, we compared our estimates to pre-pandemic literature in both healthcare and general populations in Peru, supporting the observed symptom increase during the early pandemic.

Sixth, the multivariable models included numerous covariates, which may have reduced statistical power to detect associations for predictors with low prevalence. Although the sample size was calculated based on a detectable effect size of 35%, the degrees of freedom required for modeling may have diminished sensitivity. Thus, non-significant associations should be interpreted cautiously.

Finally, some estimates showed wide confidence intervals due to low cell counts in certain exposure categories, which may affect the stability of these specific PR estimates. These results should therefore be interpreted with caution.

Despite these limitations, this preliminary study provides novel information using validated instruments to obtain a variety of characteristics that may trigger mental problems. Our findings can serve to lay the foundations for the development of new, better-designed research, which will ultimately drive the design of suitable preventive strategies. Another strength of the study is that it has captured a large and diverse sample from the two largest health systems in Peru. Finally, as mentioned in the Instruments, the Anxiety and Depression questionnaires only provide information about symptoms in patients, not a psychiatric diagnosis. Therefore, the results provided should be taken with caution.

## Conclusions

In conclusion, the results of our study reveal a worrying prevalence of depression and anxiety among health personnel at the Social Security hospitals of Piura and Lambayeque during the COVID-19 pandemic. Additionally, personal, psychosocial and work factors have been found associated with mental health outcomes such as insomnia, fear of contagion and stigmatization. These findings highlight the urgent need to take action to address the mental health of these professionals, who play a fundamental role in the response and care to the pandemic.

## Data Availability

The raw data supporting the conclusions of this article will be made available by the authors, without undue reservation.
