# Supplementary material for: Depression and anxiety in health human resources during the first COVID-19 wave in northern Peru: a multicenter study
Source: Front Psychiatry. 2025 Dec 12;16:1616381. doi: 10.3389/fpsyt.2025.1616381 (PMC12741852; doi:10.3389/fpsyt.2025.1616381)
Supplement: Supplementary file 1 [file Table1.docx]

**Material suppelmenterial**

| **Table 01.** Personal, physical, occupational and psychosocial characteristics of health personnel from three Peruvian Social Security hospitals during the COVID-19 pandemic, 2020. (N=182) | | |
| --- | --- | --- |
|  |  |  |
| **Characteristics** | | **n (%)** |
| Age (years)* | | 38.9 (23-69) |
| Sex | |  |
|  | Femele | 114 (62.6) |
|  | Male | 68 (37.4) |
| Marital status | |  |
|  | Single | 69 (37.9) |
|  | Married | 81 (44.5) |
|  | Cohabitant | 22 (12.1) |
|  | Widower | 3 (1.7) |
|  | Divorced | 7 (3.9) |
| Religion | |  |
|  | None | 8 (4.4) |
|  | Catholic | 162 (89.0) |
|  | Not Catholic | 12 (6.6) |
| Possession of children | |  |
|  | No | 62 (34.1) |
|  | Yes | 120 (65.9) |
| Role of father/mother in family | |  |
|  | No | 60 (33.0) |
|  | Yes | 122 (67.0) |
| Arterial hypertension | |  |
|  | No | 172 (94.5) |
|  | Yes | 10 (5.5) |
| Diabetes mellitus | |  |
|  | No | 177 (97.3) |
|  | Yes | 5 (2.8) |
| Obesity | |  |
|  | No | 160 (87.9) |
|  | Yes | 22 (12.1) |
| COVID-19 diagnosis | |  |
|  | No | 111 (66.1) |
|  | Yes | 57 (33.9) |
| Hospital | |  |
|  | Hospital Luis Heysen Incháustegui- Lambayeque | 50 (27.5) |
|  | Hospital Nacional Almanzor Aguinaga Asenjo-Lambayeque | 44 (24.2) |
|  | Hospital Regional José Cayetano Heredia-Piura | 88 (48.4) |
| Profession | |  |
|  | Doctor | 88 (48.4) |
|  | Nurse | 40 (22.0) |
|  | Obstetrician | 1 (0.6) |
|  | Biologist | 1 (0.6) |
|  | Dentist | 5 (2.8) |
|  | Nursign technician | 29 (15.9) |
|  | Medical technologist | 9 (5.0) |
|  | Psychologistt | 3 (1.7) |
|  | Other | 6 (3.3) |
| Economic income in MLW | |  |
|  | 1 to 3 | 21 (11.5) |
|  | 3 to 5 | 37 (20.3) |
|  | 5 to 7 | 46 (25.3) |
|  | 7 to 10 | 45 (24.7) |
|  | > 10 | 33 (18.1) |
| Role in service | |  |
|  | Leader | 23 (12.6) |
|  | Leader support | 26 (14.3) |
|  | Operation staff | 133 (73.1) |
| Type of contract | |  |
|  | Appointed/Fixed term | 74 (40.7) |
|  | Administrative services contract | 95 (52.2) |
|  | Third party service | 2 (1.1) |
|  | Resident | 1 (0.6) |
|  | Other | 10 (5.5) |
| First line of COVID-19 care | |  |
|  | No | 83 (45.6) |
|  | Yes | 99 (54.4) |
| Compliance with confinement measures | |  |
|  | Yes | 141 (77.5) |
|  | No | 41 (22.5) |
| Compliance with distancing measures | |  |
|  | Yes | 161 (88.5) |
|  | No | 21 (11.5) |
| Severity of pandemic | |  |
|  | Mild/Serious | 31 (17.0) |
|  | Very serious | 151 (83.0) |
| Confidence in government capacity to manage pandemic | |  |
|  | No | 148 (81.3) |
|  | Yes | 34 (18.7) |
| History of previous mental illness | |  |
|  | No | 176 (96.7) |
|  | Yes | 6 (3.3) |
| Family members with COVID-19 | |  |
|  | No | 76 (41.8) |
|  | Yes | 106 (58.2) |
| Family member decesased from COVID-19 | |  |
|  | No | 122 (67.0) |
|  | Yes | 60 (33.0) |
| Experience of mistreatment in health care during COVID-19 | |  |
|  | No | 97 (53.3) |
|  | Yes | 85 (46.7) |
| Concern about infecting family members | |  |
|  | Not at all/ a little | 20 (11.0) |
|  | Moderate | 21 (11.5) |
|  | A lot/ Extreme | 141 (77.5) |
| Concern about getting infected | |  |
|  | Not at all/ a little | 36 (19.8) |
|  | Moderate | 36 (19.8) |
|  | A lot/ Extreme | 110 (60.4) |
| Concern about availability of PPE | |  |
|  | Not at all/ a little | 41 (22.5) |
|  | Moderate | 45 (24.7) |
|  | A lot/ Extreme | 96 (52.8) |
| Concern about not having health insurance | |  |
|  | Not at all/ a little | 83 (45.6) |
|  | Moderate | 23 (12.6) |
|  | A lot/ Extreme | 76 (41.8) |
| Concern about working in a COVID Environment | |  |
|  | Not at all/ a little | 59 (32.4) |
|  | Moderate | 50 (27.5) |
|  | A lot/ Extreme | 73 (40.1) |
| Concern about being marginalized by family environment | |  |
|  | Not at all/ a little | 160 (87.9) |
|  | Moderate | 13 (7.1) |
|  | A lot/ Extreme | 9 (5.0) |
| Concern about being marginalized by the neighborhood | |  |
|  | Not at all/ a little | 156 (85.7) |
|  | Moderate | 17 (9.3) |
|  | A lot/ Extreme | 9 (5.0) |
| Concern about being an asymptomatic patient | |  |
|  | Not at all/ a little | 82 (45.1) |
|  | Moderate | 29 (15.9) |
|  | A lot/ Extreme | 71 (39.0) |
| Resilience | |  |
|  | Low | 43 (23.6) |
|  | High | 139 (76.4) |
| Insomnia | |  |
|  | No | 140 (77.4) |
|  | Subclinical | 38 (21.0) |
|  | Moderate clinical | 1 (0.6) |
|  | Severe clinical | 2 (1.1) |
| Physical activity level | |  |
|  | Low | 87 (47.8) |
|  | Moderate | 17 (9.3) |
|  | High | 78 (42.9) |
| Burnout syndrome | |  |
|  | No | 169 (92.9) |
|  | Yes | 13 (7.1) |
| Eating disorder | |  |
|  | No | 170 (93.9) |
|  | Yes | 11 (6.1) |
| Risk of alcohol consumption | |  |
|  | Low | 173 (96.7) |
|  | Moderate | 6 (3.4) |
|  | High | 0 (0.0) |
| Risk of tobacco use | |  |
|  | Low | 169 (93.9) |
|  | Moderate | 11 (6.1) |
|  | High | 0 (0.0) |
| Depression | |  |
|  | Minimun | 104 (57.1) |
|  | Mild | 58 (31.9) |
|  | Moderate | 17 (9.3) |
|  | Severe | 3 (1.7) |
| Anxiety | |  |
|  | No | 90 (49.5) |
|  | Mild | 68 (37.4) |
|  | Moderate | 11 (6.0) |
|  | Severe | 13 (7.1) |
| *Median (Minimun - Maximun value) | | |
| MLW: Minimun living wage | | |
| PPE: Personal Protective Equipment | | |
| Values are expressed as n (%) unless otherwise indicated. Percentages were calculated using the total N = 182 as denominator. | | |

| **Table 02.** Factors associated with depressive and anxious symptoms in health personnel of three hospitals of the Social Security of Peru, during the pandemic of COVID-19, 2020, in bivariate analysis. (N=182) | | | | | | | | |
| --- | --- | --- | --- | --- | --- | --- | --- | --- |
| Variables | | Depression | | p* | Anxiety | | p* |  |
|  |  | No (n=104) | Yes (n=78) |  | No (n=90) | Yes (n=92) |  |  |
|  |  | n(%) | n(%) |  | n(%) | n(%) |  |  |
| Age (years)* | | 37 (23-69) | 35 (25-66) | 0.129 | 36.5 (24-69) | 35.5 (23-68) | 0.114 |  |
| Sex | |  |  | **0.027** |  |  | **0.024** |  |
|  | Female | 58 (50.9) | 56 (49.1) |  | 49 (43.0) | 65 (57.0) |  |  |
|  | Male | 46 (67.7) | 22 (32.4) |  | 41 (60.3) | 27 (39.7) |  |  |
| Single | |  |  | 0.094 |  |  | 0.517 |  |
|  | No | 70 (62.0) | 43 (38.1) |  | 58 (51.3) | 55 (48.7) |  |  |
|  | Yes | 34 (49.3) | 35 (50.7) |  | 32 (46.4) | 37 (53.6) |  |  |
| Arterial hypertension | |  |  | 0.639 |  |  | 0.539 |  |
|  | No | 99 (57.6) | 73 (42.4) |  | 86 (50.0) | 86 (50.0) |  |  |
|  | Yes | 5 (50.0) | 5 (50.0) |  | 4 (40.0) | 6 (60.0) |  |  |
| Diabetes | |  |  | 0.432 |  |  | 0.166 |  |
|  | No | 102 (57.6) | 75 (42.4) |  | 86 (48.6) | 91 (51.4) |  |  |
|  | Yes | 2 (40.0) | 3 (60.0) |  | 4 (80.0) | 1 (20.0) |  |  |
| Obesity | |  |  | 0.793 |  |  | 0.335 |  |
|  | No | 92 (57.5) | 68 (42.5) |  | 77 (48.1) | 83 (51.9) |  |  |
|  | Yes | 12 (54.6) | 10 (45.5) |  | 13 (59.1) | 9 (40.9) |  |  |
| COVID-19 diagnosis | |  |  | **0.024** |  |  | 0.058 |  |
|  | No | 69 (62.2) | 42 (37.8) |  | 60 (54.1) | 51 (46.0) |  |  |
|  | Yes | 25 (43.9) | 32 (56.1) |  | 22 (38.6) | 35 (61.4) |  |  |
| Catholic religion | |  |  | 0.452 |  |  | 0.599 |  |
|  | No | 13 (65.0) | 7 (35.0) |  | 11 (55.0) | 9 (45.0) |  |  |
|  | Yes | 91 (56.2) | 71 (43.9) |  | 79 (48.8) | 83 (51.2) |  |  |
| Possession of children | |  |  | 0.443 |  |  | 0.837 |  |
|  | No | 33 (53.2) | 29 (46.7) |  | 30 (48.4) | 32 (51.6) |  |  |
|  | Yes | 71 (59.2) | 49 (40.8) |  | 60 (50.0) | 60 (50.0) |  |  |
| Role of father/mother in family | |  |  | 0.295 |  |  | 0.400 |  |
|  | No | 31 (51.7) | 29 (48.3) |  | 27 (45.0) | 33 (55.0) |  |  |
|  | Yes | 73 (59.8) | 49 (40.2) |  | 63 (51.6) | 59 (48.4) |  |  |
| History of previous mental illness | |  |  | 0.231 |  |  | 0.978 |  |
|  | No | 102 (58.0) | 74 (42.1) |  | 87 (49.4) | 89 (50.6) |  |  |
|  | Yes | 2 (33.3) | 4 (66.7) |  | 3 (50.0) | 3 (50.0) |  |  |
| Family members with COVID-19 | |  |  | **0.021** |  |  | 0.184 |  |
|  | No | 51 (67.1) | 25 (32.9) |  | 42 (55.3) | 34 (44.7) |  |  |
|  | Yes | 53 (50.0) | 53 (50.0) |  | 48 (45.3) | 58 (54.7) |  |  |
| Family member decesased from COVID-19 | |  |  | 0.092 |  |  | 0.917 |  |
|  | No | 75 (61.5) | 47 (38.5) |  | 60 (49.2) | 62 (50.8) |  |  |
|  | Yes | 29 (48.3) | 31 (51.7) |  | 30 (50.0) | 30 (50.0) |  |  |
| Economic income in MLW | |  |  | 0.312 |  |  | 0.467 |  |
|  | 1 to 3 | 9 (42.9) | 12 (57.1) |  | 9 (42.9) | 12 (57.1) |  |  |
|  | 3 to 5 | 23 (62.2) | 14 (37.8) |  | 17 (46.0) | 20 (54.1) |  |  |
|  | 5 to 7 | 30 (65.2) | 16 (34.8) |  | 25 (54.4) | 21 (45.7) |  |  |
|  | 7 to 10 | 22 (48.9) | 23 (51.1) |  | 19 (42.2) | 26 (57.8) |  |  |
|  | > 10 | 20 (60.6) | 13 (39.4) |  | 20 (60.6) | 13 (39.4) |  |  |
| Doctor | |  |  | 0.266 |  |  | 0.104 |  |
|  | No | 50 (53.2) | 44 (46.8) |  | 41 (43.6) | 53 (56.4) |  |  |
|  | Yes | 54 (61.4) | 34 (38.6) |  | 49 (55.7) | 39 (44.3) |  |  |
| Role in service | |  |  | 0.684 |  |  | 0.744 |  |
|  | Leader | 15 (65.2) | 8 (34.9) |  | 13 (56.5) | 10 (43.5) |  |  |
|  | Leader support | 14 (53.9) | 12 (46.2) |  | 12 (46.2) | 14 (53.9) |  |  |
|  | Operation staff | 75 (56.4) | 58 (43.6) |  | 65 (48.9) | 68 (51.1) |  |  |
| Appointed/Fixed term | |  |  | 0.408 |  |  | 0.304 |  |
|  | No | 59 (54.6) | 49 (45.4) |  | 50 (46.3) | 58 (53.7) |  |  |
|  | Yes | 45 (60.8) | 29 (39.2) |  | 40 (54.1) | 34 (46.0) |  |  |
| Compliance with confinement measures | |  |  | **0.046** |  |  | 0.540 |  |
|  | Yes | 75 (53.2) | 66 (46.8) |  | 68 (48.2) | 73 (51.8) |  |  |
|  | No | 29 (70.7) | 12 (29.3) |  | 22 (53.7) | 19 (46.3) |  |  |
| Compliance with distancing measures | |  |  | 0.160 |  |  | **0.012** |  |
|  | Yes | 95 (59.0) | 66 (41.0) |  | 85 (52.8) | 76 (47.2) |  |  |
|  | No | 9 (42.9) | 12 (57.1) |  | 5 (23.8) | 16 (76.2) |  |  |
| Severity of pandemic | |  |  | 0.190 |  |  | 0.292 |  |
|  | Mild/Serious | 21 (67.7) | 10 (32.3) |  | 18 (58.1) | 13 (41.9) |  |  |
|  | Very serious | 83 (55.0) | 68 (45.0) |  | 72 (47.7) | 79 (52.3) |  |  |
| Confidence in government capacity to manage pandemic | |  |  | 0.826 |  |  | 0.406 |  |
|  | No | 84 (56.8) | 64 (43.2) |  | 71 (48.0) | 77 (52.0) |  |  |
|  | Yes | 20 (58.8) | 14 (41.2) |  | 19 (55.9) | 15 (44.1) |  |  |
| Experience of mistreatment in health care during COVID-19 | |  |  | **0.001** |  |  | **0.037** |  |
|  | No | 67 (69.1) | 30 (30.9) |  | 55 (56.7) | 42 (43.3) |  |  |
|  | Yes | 37 (43.5) | 48 (56.5) |  | 35 (41.2) | 50 (58.8) |  |  |
| Concern about infecting family members | |  |  | **0.027** |  |  | **0.001** |  |
|  | Not at all/ a little | 17 (85.0) | 3 (15.0) |  | 16 (80.0) | 4 (20.0) |  |  |
|  | Moderate | 12 (57.1) | 9 (42.9) |  | 15 (71.4) | 6 (28.6) |  |  |
|  | A lot/ Extreme | 75 (53.2) | 66 (46.8) |  | 59 (41.8) | 82 (58.2) |  |  |
| Concern about becoming infected | |  |  | 0.840 |  |  | 0.264 |  |
|  | Not at all/ a little | 21 (58.3) | 15 (41.7) |  | 22 (61.1) | 14 (38.9) |  |  |
|  | Moderate | 19 (52.8) | 17 (47.2) |  | 18 (50.0) | 18 (50.0) |  |  |
|  | A lot/ Extreme | 64 (58.2) | 46 (41.8) |  | 50 (45.5) | 60 (54.5) |  |  |
| Concern about availability of PPE | |  |  | 0.129 |  |  | **0.021** |  |
|  | Not at all/ a little | 28 (68.3) | 13 (31.7) |  | 28 (68.3) | 13 (31.7) |  |  |
|  | Moderate | 21 (46.7) | 24 (53.3) |  | 21 (46.7) | 24 (53.3) |  |  |
|  | A lot/ Extreme | 55 (57.3) | 41 (42.7) |  | 41 (42.7) | 55 (57.3) |  |  |
| Concern about not having health insurance | |  |  | 0.340 |  |  | 0.958 |  |
|  | Not at all/ a little | 52 (62.7) | 31 (37.4) |  | 42 (50.6) | 41 (49.4) |  |  |
|  | Moderate | 11 (47.8) | 12 (52.2) |  | 11 (47.8) | 12 (52.2) |  |  |
|  | A lot/ Extreme | 41 (54.0) | 35 (46.1) |  | 37 (48.7) | 39 (51.3) |  |  |
| Concern about working in a COVID Environment | |  |  | 0.107 |  |  | **<0.001** |  |
|  | Not at all/ a little | 39 (66.1) | 20 (33.9) |  | 43 (72.9) | 16 (27.1) |  |  |
|  | Moderate | 23 (46.0) | 27 (54.0) |  | 22 (44.0) | 28 (56.0) |  |  |
|  | A lot/ Extreme | 42 (57.5) | 31 (42.5) |  | 25 (34.3) | 48 (65.8) |  |  |
| Concern about being marginalized by family environment | |  |  | 0.700 |  |  | 0.209 |  |
|  | Not at all/ a little | 93 (58.1) | 67 (41.9) |  | 83 (51.9) | 77 (48.1) |  |  |
|  | Moderate | 7 (53.9) | 6 (46.2) |  | 4 (30.8) | 9 (69.2) |  |  |
|  | A lot/ Extreme | 4 (44.4) | 5 (55.6) |  | 3 (33.3) | 6 (66.7) |  |  |
| Concern about being marginalized by the neighborhood | |  |  | 0.055 |  |  | **0.010** |  |
|  | Not at all/ a little | 94 (60.3) | 62 (39.7) |  | 84 (53.9) | 72 (46.2) |  |  |
|  | Moderate | 8 (47.1) | 9 (52.9) |  | 5 (29.4) | 12 (70.6) |  |  |
|  | A lot/ Extreme | 2 (22.2) | 7 (77.8) |  | 1 (11.1) | 8 (88.9) |  |  |
| Concern about being an asymptomatic patient | |  |  | **0.012** |  |  | **<0.001** |  |
|  | Not at all/ a little | 55 (67.1) | 27 (32.9) |  | 57 (69.5) | 25 (30.5) |  |  |
|  | Moderate | 18 (62.1) | 11 (37.9) |  | 13 (44.8) | 16 (55.2) |  |  |
|  | A lot/ Extreme | 31 (43.7) | 40 (56.3) |  | 20 (28.2) | 51 (71.8) |  |  |
| Resilience | |  |  | 0.208 |  |  | 0.255 |  |
|  | Low | 21 (48.8) | 22 (51.2) |  | 18 (41.9) | 25 (58.1) |  |  |
|  | High | 83 (59.7) | 56 (40.3) |  | 72 (51.8) | 67 (48.2) |  |  |
| Insomnia | |  |  | **<0.001** |  |  | **0.029** |  |
|  | No | 92 (65.7) | 48 (34.3) |  | 75 (53.6) | 65 (46.4) |  |  |
|  | Yes | 11 (26.8) | 30 (73.2) |  | 14 (34.2) | 27 (65.9) |  |  |
| Physical activity level | |  |  | **0.045** |  |  | 0.365 |  |
|  | Low | 54 (62.1) | 33 (37.9) |  | 42 (48.3) | 45 (51.7) |  |  |
|  | Moderate | 5 (29.4) | 12 (70.6) |  | 6 (35.3) | 11 (64.7) |  |  |
|  | High | 45 (57.7) | 33 (42.3) |  | 42 (53.9) | 36 (46.2) |  |  |
| Burnout syndrome | |  |  | **<0.001** |  |  | 0.162 |  |
|  | No | 103 (61.0) | 66 (39.1) |  | 86 (50.9) | 83 (49.1) |  |  |
|  | Yes | 1 (7.7) | 12 (92.3) |  | 4 (30.8) | 9 (69.2) |  |  |
| Eating disorder | |  |  | 0.532** |  |  | 0.360 |  |
|  | No | 99 (58.2) | 71 (41.8) |  | 86 (50.6) | 84 (49.4) |  |  |
|  | Yes | 5 (45.5) | 6 (54.6) |  | 4 (36.4) | 7 (63.6) |  |  |
| Risk of alcohol consumption | |  |  | 0.405** |  |  | 0.999** |  |
|  | Low | 100 (57.8) | 73 (42.2) |  | 85 (49.1) | 88 (50.9) |  |  |
|  | Moderate | 2 (33.3) | 4 (66.7) |  | 3 (50.0) | 3 (50.0) |  |  |
| Risk of tobacco use | |  |  | 0.759** |  |  | 0.103 |  |
|  | Low | 95 (56.2) | 74 (43.8) |  | 80 (47.3) | 89 (52.7) |  |  |
|  | Moderate | 7 (63.6) | 4 (36.4) |  | 8 (72.7) | 3 (27.3) |  |  |
| * p-value of categorical variables calculated with the Chi-Square test. | | | | | | | | |
| ** p-value of categorical variables calculated with Fisher's exact test. | | | | | | | | |
| ¶ p-value of categorical-numerical variables calculated with the U-test (Mann-Whitney). | | | | | | | | |
| Ɨ Median - interquartile range | | | | | | | | |
| MLW: Minimun living wage | | | | | | | | |
| PPE: Personal Protective Equipment | | | | | | | | |

| **Table 03**. Factors associated with depressive and anxious symptoms in health personnel of three hospitals of the Social Security of Peru, during the pandemic of COVID-19, 2020, in simple and multiple regression analysis. | | | | | | | | | | | | | | | | |
| --- | --- | --- | --- | --- | --- | --- | --- | --- | --- | --- | --- | --- | --- | --- | --- | --- |
|  |  |  |  |  | |  |  |  | |  |  |  | |  |  |  |
| **Characteristics** | | **Depression** | | | | | | | **Anxiety** | | | | | | | |
|  |  | **Simple regression** | | | **Multiple regression** | | | | **Simple regression** | | | | **Multiple regression** | | | |
|  |  | **PR** | **IC 95%** | **p*** | | **PR** | **IC 95%** | **p*** | | **PR** | **IC 95%** | **p*** | | **PR** | **IC 95%** | **p*** |
| Age (years)* | | 0.99 | 0.97-0.99 | **<0.001** | | 0.99 | 0.98-0.99 | **<0.001** | | 0.99 | 0.97-1.00 | 0.184 | |  |  |  |
| Sex | |  |  |  | |  |  |  | |  |  |  | |  |  |  |
|  | Female | Ref. |  |  | |  |  |  | | Ref. |  |  | | Ref. |  |  |
|  | Male | 0.66 | 0.34-1.26 | 0.208 | |  |  |  | | 0.70 | 0.62-0.78 | **<0.001** | | 0.83 | 0.68-1.01 | 0.062 |
| Single | |  |  |  | |  |  |  | |  |  |  | |  |  |  |
|  | No | Ref. |  |  | |  |  |  | | Ref. |  |  | |  |  |  |
|  | Yes | 1.33 | 0.91-1.94 | 0.135 | |  |  |  | | 1.10 | 0.89-1.36 | 0.361 | |  |  |  |
| Arterial hypertension | |  |  |  | |  |  |  | |  |  |  | |  |  |  |
|  | No | Ref. |  |  | |  |  |  | | Ref. |  |  | |  |  |  |
|  | Yes | 1.18 | 0.68-2.06 | 0.564 | |  |  |  | | 1.20 | 0.54-2.66 | 0.653 | |  |  |  |
| Diabetes | |  |  |  | |  |  |  | |  |  |  | |  |  |  |
|  | No | Ref. |  |  | | Ref. |  |  | | Ref. |  |  | |  |  |  |
|  | Yes | 1.42 | 1.08-1.85 | **0.011** | | 1.41 | 1.01-1.99 | **0.048** | | 0.39 | 0.04-4.11 | 0.432 | |  |  |  |
| Obesity | |  |  |  | |  |  |  | |  |  |  | |  |  |  |
|  | No | Ref. |  |  | |  |  |  | | Ref. |  |  | |  |  |  |
|  | Yes | 1.07 | 0.63-1.81 | 0.802 | |  |  |  | | 0.79 | 0.41-1.53 | 0.482 | |  |  |  |
| COVID-19 diagnosis | |  |  |  | |  |  |  | |  |  |  | |  |  |  |
|  | No | Ref. |  |  | | Ref. |  |  | | Ref. |  |  | |  |  |  |
|  | Yes | 1.48 | 1.17-1.8 | **0.001** | | 1.22 | 0.84-1.78 | 0.298 | | 1.34 | 0.87-2.06 | 0.190 | |  |  |  |
| Catholic religion | |  |  |  | |  |  |  | |  |  |  | |  |  |  |
|  | No | Ref. |  |  | |  |  |  | | Ref. |  |  | |  |  |  |
|  | Yes | 1.25 | 0.52-3.02 | 0.617 | |  |  |  | | 1.14 | 0.64-2.04 | 0.662 | |  |  |  |
| Possession of children | |  |  |  | |  |  |  | |  |  |  | |  |  |  |
|  | No | Ref. |  |  | |  |  |  | | Ref. |  |  | |  |  |  |
|  | Yes | 0.87 | 0.64-1.19 | 0.395 | |  |  |  | | 0.97 | 0.86-1.09 | 0.586 | |  |  |  |
| Role of father/mother in family | |  |  |  | |  |  |  | |  |  |  | |  |  |  |
|  | No | Ref. |  |  | |  |  |  | | Ref. |  |  | |  |  |  |
|  | Yes | 0.83 | 0.55-1.26 | 0.379 | |  |  |  | | 0.88 | 0.74-1.05 | 0.144 | |  |  |  |
| History of previous mental illness | |  |  |  | |  |  |  | |  |  |  | |  |  |  |
|  | No | Ref. |  |  | | Ref. |  |  | | Ref. |  |  | |  |  |  |
|  | Yes | 1.59 | 1.31-1.92 | **<0.001** | | 1.32 | 1.13-1.55 | **0.001** | | 0.99 | 0.49-1.99 | 0.975 | |  |  |  |
| Family members with COVID-19 | |  |  |  | |  |  |  | |  |  |  | |  |  |  |
|  | No | Ref. |  |  | | Ref. |  |  | | Ref. |  |  | | Ref. |  |  |
|  | Yes | 1.52 | 1.27-1.81 | **<0.001** | | 1.05 | 0.93-1.20 | 0.425 | | 1.22 | 1.06-1.41 | **0.005** | | 1.04 | 1.01-1.07 | **0.004** |
| Family member decesased from COVID-19 | |  |  |  | |  |  |  | |  |  |  | |  |  |  |
|  | No | Ref. |  |  | |  |  |  | | Ref. |  |  | |  |  |  |
|  | Yes | 1.34 | 0.95-1.90 | 0.100 | |  |  |  | | 0.98 | 0.70-1.38 | 0.925 | |  |  |  |
| Economic income in MLW | |  |  |  | |  |  |  | |  |  |  | |  |  |  |
|  | 1 to 3 | Ref. |  |  | |  |  |  | | Ref. |  |  | |  |  |  |
|  | 3 to 5 | 0.66 | 0.25-1.76 | 0.407 | |  |  |  | | 0.95 | 0.61-1.46 | 0.801 | |  |  |  |
|  | 5 to 7 | 0.61 | 0.34-1.09 | 0.094 | |  |  |  | | 0.80 | 0.55-1.16 | 0.239 | |  |  |  |
|  | 7 to 10 | 0.89 | 0.51-1.56 | 0.695 | |  |  |  | | 1.01 | 0.79-1.30 | 0.931 | |  |  |  |
|  | > 10 | 0.69 | 0.32-1.51 | 0.352 | |  |  |  | | 0.69 | 0.44-1.07 | 0.098 | |  |  |  |
| Doctor | |  |  |  | |  |  |  | |  |  |  | |  |  |  |
|  | No | Ref. |  |  | |  |  |  | | Ref. |  |  | |  |  |  |
|  | Yes | 0.83 | 0.44-1.54 | 0.548 | |  |  |  | | 0.79 | 0.58-1.07 | 0.124 | |  |  |  |
| Role in service | |  |  |  | |  |  |  | |  |  |  | |  |  |  |
|  | Leader | Ref. |  |  | |  |  |  | | Ref. |  |  | |  |  |  |
|  | Leader support | 1.33 | 0.67-2.64 | 0.420 | |  |  |  | | 1.24 | 0.90-1.70 | 0.188 | |  |  |  |
|  | Operation staff | 1.25 | 0.90-1.75 | 0.181 | |  |  |  | | 1.18 | 0.87-1.59 | 0.296 | |  |  |  |
| Appointed/Fixed term | |  |  |  | |  |  |  | |  |  |  | |  |  |  |
|  | No | Ref. |  |  | |  |  |  | | Ref. |  |  | | Ref. |  |  |
|  | Yes | 0.86 | 0.52-1.43 | 0.569 | |  |  |  | | 0.86 | 0.79-0.93 | **<0.001** | | 0.89 | 0.74-1.06 | 0.193 |
| Compliance with confinement measures | |  |  |  | |  |  |  | |  |  |  | |  |  |  |
|  | Yes | Ref. |  |  | |  |  |  | | Ref. |  |  | |  |  |  |
|  | No | 0.63 | 0.45-0.88 | 0.007 | |  |  |  | | 0.90 | 0.76-1.06 | 0.195 | |  |  |  |
| Compliance with distancing measures | |  |  |  | |  |  |  | |  |  |  | |  |  |  |
|  | Yes | Ref. |  |  | |  |  |  | | Ref. |  |  | |  |  |  |
|  | No | 1.39 | 0.69-2.80 | 0.351 | |  |  |  | | 1.61 | 0.97-2.70 | 0.068 | |  |  |  |
| Severity of pandemic | |  |  |  | |  |  |  | |  |  |  | |  |  |  |
|  | Mild/Serious | Ref. |  |  | |  |  |  | | Ref. |  |  | |  |  |  |
|  | Very serious | 1.40 | 0.79-2.45 | 0.246 | |  |  |  | | 1.25 | 0.75-2.09 | 0.400 | |  |  |  |
| Confidence in goverment capacity to manage pandemic | |  |  |  | |  |  |  | |  |  |  | |  |  |  |
|  | No | Ref. |  |  | |  |  |  | | Ref. |  |  | |  |  |  |
|  | Yes | 0.95 | 0.76-1.20 | 0.677 | |  |  |  | | 0.85 | 0.62-1.16 | 0.299 | |  |  |  |
| Experience of mistreatment in health care during COVID-19 | |  |  |  | |  |  |  | |  |  |  | |  |  |  |
|  | No | Ref. |  |  | | Ref. |  |  | | Ref. |  |  | | Ref. |  |  |
|  | Yes | 1.83 | 1.46-2.29 | **<0.001** | | 1.35 | 1.01-1.82 | **0.048** | | 1.36 | 1.04-1.78 | **0.025** | | 1.05 | 0.79-1.38 | 0.741 |
| Concern about infecting family members | |  |  |  | |  |  |  | |  |  |  | |  |  |  |
|  | Not at all/ a little | Ref. |  |  | | Ref. |  |  | | Ref. |  |  | |  |  |  |
|  | Moderate | 2.86 | 1.33-6.15 | **0.007** | | 2.45 | 0.77-7.86 | 0.131 | | 1.43 | 0.67-3.03 | 0.351 | |  |  |  |
|  | A lot/ Extreme | 3.12 | 1.12-8.71 | **0.030** | | 1.83 | 0.61-5.48 | 0.283 | | 2.91 | 0.91-9.30 | 0.072 | |  |  |  |
| Concern about becoming infected | |  |  |  | |  |  |  | |  |  |  | |  |  |  |
|  | Not at all/ a little | Ref. |  |  | |  |  |  | | Ref. |  |  | | Ref. |  |  |
|  | Moderate | 1.13 | 0.91-1.42 | 0.274 | |  |  |  | | 1.29 | 1.05-1.58 | **0.016** | | 0.65 | 0.37-1.15 | 0.137 |
|  | A lot/ Extreme | 1.00 | 0.85-1.19 | 0.967 | |  |  |  | | 1.40 | 0.92-2.13 | 0.112 | | 0.65 | 0.35-1.19 | 0.161 |
| Concern about availability of PPE | |  |  |  | |  |  |  | |  |  |  | |  |  |  |
|  | Not at all/ a little | Ref. |  |  | |  |  |  | | Ref. |  |  | | Ref. |  |  |
|  | Moderate | 1.68 | 0.99-2.87 | 0.056 | |  |  |  | | 1.68 | 1.36-2.07 | **<0.001** | | 1.16 | 0.96-1-40 | 0.131 |
|  | A lot/ Extreme | 1.35 | 0.93-1.95 | 0.116 | |  |  |  | | 1.81 | 1.70-1.92 | **<0.001** | | 1.20 | 1.01-1.44 | **0.047** |
| Concern about not having health insurance | |  |  |  | |  |  |  | |  |  |  | |  |  |  |
|  | Not at all/ a little | Ref. |  |  | | Ref. |  |  | | Ref. |  |  | |  |  |  |
|  | Moderate | 1.40 | 1.09-1.79 | **0.008** | | 1.16 | 0.90-1.50 | 0.251 | | 1.06 | 0.92-1.21 | 0.441 | |  |  |  |
|  | A lot/ Extreme | 1.23 | 0.73-2.10 | 0.439 | | 1.19 | 0.72-1.73 | 0.612 | | 1.04 | 0.70-1.55 | 0.853 | |  |  |  |
| Concern about working in a COVID Environment | |  |  |  | |  |  |  | |  |  |  | |  |  |  |
|  | Not at all/ a little | Ref. |  |  | | Ref. |  |  | | Ref. |  |  | | Ref. |  |  |
|  | Moderate | 1.59 | 1.19-2.13 | **0.002** | | 1.23 | 1.07-1.41 | **0.004** | | 2.07 | 1.89-2.25 | **<0.001** | | 1.92 | 1.49-2.47 | **<0.001** |
|  | A lot/ Extreme | 1.25 | 0.91-1.72 | 0.910 | | 1.23 | 1.12-1.35 | **<0.001** | | 2.42 | 1.80-3.26 | **<0.001** | | 2.25 | 1.42-3.59 | **0.001** |
| Concern about being marginalized by family environment | |  |  |  | |  |  |  | |  |  |  | |  |  |  |
|  | Not at all/ a little | Ref. |  |  | |  |  |  | | Ref. |  |  | |  |  |  |
|  | Moderate | 1.10 | 0.73-1.67 | 0.644 | |  |  |  | | 1.44 | 0.89-2.32 | 0.136 | |  |  |  |
|  | A lot/ Extreme | 1.33 | 0.70-2.52 | 0.387 | |  |  |  | | 1.39 | 0.93-2.05 | 0.105 | |  |  |  |
| Concern about being marginalized by the neighborhood | |  |  |  | |  |  |  | |  |  |  | |  |  |  |
|  | Not at all/ a little | Ref. |  |  | | Ref. |  |  | | Ref. |  |  | | Ref. |  |  |
|  | Moderate | 1.33 | 0.87-2.03 | 0.183 | | 0.96 | 0.60-1.53 | 0.853 | | 1.53 | 1.38-1.70 | **<0.001** | | 1.26 | 1.02-1.46 | **0.035** |
|  | A lot/ Extreme | 1.96 | 1.88-2.03 | **<0.001** | | 2.00 | 1.38-2.91 | **<0.001** | | 1.93 | 1.60-2.32 | **<0.001** | | 1.85 | 1.42-3.59 | **0.001** |
| Concern about being an asymptomatic patient | |  |  |  | |  |  |  | |  |  |  | |  |  |  |
|  | Not at all/ a little | Ref. |  |  | |  |  |  | | Ref. |  |  | | Ref. |  |  |
|  | Moderate | 1.15 | 0.77-1.72 | 0.490 | |  |  |  | | 1.81 | 0.85-3.84 | 0.123 | | 1.48 | 0.71-3.11 | 0.298 |
|  | A lot/ Extreme | 1.71 | 0.89-3.28 | 0.106 | |  |  |  | | 2.36 | 1.29-4.31 | **0.005** | | 1.79 | 0.92-3.47 | 0.087 |
| Resilience | |  |  |  | |  |  |  | |  |  |  | |  |  |  |
|  | Low | Ref. |  |  | |  |  |  | | Ref. |  |  | |  |  |  |
|  | High | 0.79 | 0.39-1.59 | 0.506 | |  |  |  | | 0.83 | 0.54-1.28 | 0.396 | |  |  |  |
| Insomnia | |  |  |  | |  |  |  | |  |  |  | |  |  |  |
|  | No | Ref. |  |  | | Ref. |  |  | | Ref. |  |  | | Ref. |  |  |
|  | Yes | 2.13 | 1.80-2.53 | **<0.001** | | 1.62 | 1.10-2.42 | **0.015** | | 1.42 | 1.02-1.98 | **0.040** | | 1.40 | 0.89-2.20 | 0.144 |
| Physical activity level | |  |  |  | |  |  |  | |  |  |  | |  |  |  |
|  | Low | Ref. |  |  | | Ref. |  |  | | Ref. |  |  | |  |  |  |
|  | Moderate | 1.86 | 1.08-3.21 | **0.025** | | 1.33 | 0.77-2.31 | 0.310 | | 1.25 | 0.49-3.19 | 0.640 | |  |  |  |
|  | High | 1.12 | 0.64-1.94 | 0.698 | | 1.19 | 0.69-2.07 | 0.541 | | 0.89 | 0.42-1.88 | 0.765 | |  |  |  |
| Burnout syndrome | |  |  |  | |  |  |  | |  |  |  | |  |  |  |
|  | No | Ref. |  |  | | Ref. |  |  | | Ref. |  |  | |  |  |  |
|  | Yes | 2.36 | 1.76-3.18 | **<0.001** | | 1.42 | 1.04-1.95 | **0.028** | | 1.41 | 0.81-2.44 | 0.221 | |  |  |  |
| Eating disorder | |  |  |  | |  |  |  | |  |  |  | |  |  |  |
|  | No | Ref. |  |  | | Ref. |  |  | | Ref. |  |  | |  |  |  |
|  | Yes | 1.31 | 1.01-1.69 | **0.042** | | 1.30 | 0.83-2.03 | 0.245 | | 1.29 | 0.72-2.30 | 0.393 | |  |  |  |
| Risk of alcohol consumption | |  |  |  | |  |  |  | |  |  |  | |  |  |  |
|  | Low | Ref. |  |  | |  |  |  | | Ref. |  |  | |  |  |  |
|  | Moderate | 0.83 | 0.39-1.78 | 0.634 | |  |  |  | | 0.98 | 0.24-3.98 | 0.981 | |  |  |  |
| Risk of tobacco use | |  |  |  | |  |  |  | |  |  |  | |  |  |  |
|  | Low | Ref. |  |  | |  |  |  | | Ref. |  |  | | Ref. |  |  |
|  | Moderate | 1.58 | 0.81-3.07 | 0.177 | |  |  |  | | 0.52 | 0.35-0.77 | 0.001 | | 0.46 | 0.40-0.52 | **<0.001** |
| *p-values obtained with Generalized Linear Models (GLM), Poisson family, log-link function, robust variance, hospital as a cluster | | | | | | | | | | | | | | | | |

| **Tabla 04.** Factors associated with depressive and anxious symptoms in health personnel of three hospitals of the Social Security of Peru, during the pandemic of COVID-19, 2020, in sensitivity multivariable analysis (cut-off ≥10, Model 2 and Model 3) | | | | | | | | | | | | | | | | | | | | | | | |  |  |  |  |  |  |  |  |  |  |  |  |  |  |  |
| --- | --- | --- | --- | --- | --- | --- | --- | --- | --- | --- | --- | --- | --- | --- | --- | --- | --- | --- | --- | --- | --- | --- | --- | --- | --- | --- | --- | --- | --- | --- | --- | --- | --- | --- | --- | --- | --- | --- |
|  |  |  |  |  |  |  |  |  |  |  |  |  |  |  | | |  | |  | |  | |  | | |  | |  | |  | |  | |  | |  | |  |
| **Características** | | ***Depression*** | | | | | | **Anxiety** | | | | | |  |  |  | |  | |  | |  | | |  | |  | |  | |  | |  | |  | |  |  |
|  |  | **Multiple regression_Model 2** | | | **Multiple regression_Model 3*** | | | **Multiple regression - Modelo 2** | | | **Regresión múltiple - Modelo 3*** | | |  |  |  | |  | |  | |  | | |  | |  | |  | |  | |  | |  | |  |  |
|  |  | **PR** | **IC 95%** | **p*** | **PR** | **IC 95%** | **p*** | **PR** | **IC 95%** | **p*** | **PR** | **IC 95%** | **p*** |  | | |  | |  | |  | |  | | |  | |  | |  | |  | |  | |  | |  |
| Age (years)* | |  |  |  |  |  |  |  |  |  | 1.01 | 0.96-1.06 | 0.639 |  | | |  | |  | |  | |  | | |  | |  | |  | |  | |  | |  | |  |
| Sex | |  |  |  |  |  |  |  |  |  |  |  |  |  | | |  | |  | |  | |  | | |  | |  | |  | |  | |  | |  | |  |
|  | Female | Ref. |  |  | Ref. |  |  |  |  |  | Ref. |  |  |  | | |  | |  | |  | |  | | |  | |  | |  | |  | |  | |  | |  |
|  | Male | 0.38 | 0.20-0.73 | 0.003 | 0.40 | 0.29-0.55 | **<0.001** |  |  |  | 0.42 | 0.20-0.88 | **0.022** |  | | |  | |  | |  | |  | | |  | |  | |  | |  | |  | |  | |  |
| Single | |  |  |  |  |  |  |  |  |  |  |  |  |  | | |  | |  | |  | |  | | |  | |  | |  | |  | |  | |  | |  |
|  | No |  |  |  |  |  |  |  |  |  |  |  |  |  | | |  | |  | |  | |  | | |  | |  | |  | |  | |  | |  | |  |
|  | Yes |  |  |  |  |  |  |  |  |  |  |  |  |  | | |  | |  | |  | |  | | |  | |  | |  | |  | |  | |  | |  |
| Arterial hypertension | |  |  |  |  |  |  |  |  |  |  |  |  |  | | |  | |  | |  | |  | | |  | |  | |  | |  | |  | |  | |  |
|  | No |  |  |  |  |  |  |  |  |  |  |  |  |  | | |  | |  | |  | |  | | |  | |  | |  | |  | |  | |  | |  |
|  | Yes |  |  |  |  |  |  |  |  |  |  |  |  |  | | |  | |  | |  | |  | | |  | |  | |  | |  | |  | |  | |  |
| Diabetes | |  |  |  |  |  |  |  |  |  |  |  |  |  | | |  | |  | |  | |  | | |  | |  | |  | |  | |  | |  | |  |
|  | No |  |  |  |  |  |  |  |  |  |  |  |  |  | | |  | |  | |  | |  | | |  | |  | |  | |  | |  | |  | |  |
|  | Yes |  |  |  |  |  |  |  |  |  |  |  |  |  | | |  | |  | |  | |  | | |  | |  | |  | |  | |  | |  | |  |
| Obesity | |  |  |  |  |  |  |  |  |  |  |  |  |  | | |  | |  | |  | |  | | |  | |  | |  | |  | |  | |  | |  |
|  | No |  |  |  |  |  |  |  |  |  |  |  |  |  | | |  | |  | |  | |  | | |  | |  | |  | |  | |  | |  | |  |
|  | Yes |  |  |  |  |  |  |  |  |  |  |  |  |  | | |  | |  | |  | |  | | |  | |  | |  | |  | |  | |  | |  |
| COVID-19 diagnosis | |  |  |  |  |  |  |  |  |  |  |  |  |  | | |  | |  | |  | |  | | |  | |  | |  | |  | |  | |  | |  |
|  | No |  |  |  |  |  |  |  |  |  |  |  |  |  | | |  | |  | |  | |  | | |  | |  | |  | |  | |  | |  | |  |
|  | Yes |  |  |  |  |  |  |  |  |  |  |  |  |  | | |  | |  | |  | |  | | |  | |  | |  | |  | |  | |  | |  |
| Catholic religion | |  |  |  |  |  |  |  |  |  |  |  |  |  | | |  | |  | |  | |  | | |  | |  | |  | |  | |  | |  | |  |
|  | No |  |  |  |  |  |  |  |  |  |  |  |  |  | | |  | |  | |  | |  | | |  | |  | |  | |  | |  | |  | |  |
|  | Yes |  |  |  |  |  |  |  |  |  |  |  |  |  | | |  | |  | |  | |  | | |  | |  | |  | |  | |  | |  | |  |
| Possession of children | |  |  |  |  |  |  |  |  |  |  |  |  |  | | |  | |  | |  | |  | | |  | |  | |  | |  | |  | |  | |  |
|  | No |  |  |  |  |  |  |  |  |  |  |  |  |  | | |  | |  | |  | |  | | |  | |  | |  | |  | |  | |  | |  |
|  | Yes |  |  |  |  |  |  |  |  |  |  |  |  |  | | |  | |  | |  | |  | | |  | |  | |  | |  | |  | |  | |  |
| Role of father/mother in family | |  |  |  |  |  |  |  |  |  |  |  |  |  | | |  | |  | |  | |  | | |  | |  | |  | |  | |  | |  | |  |
|  | No |  |  |  |  |  |  |  |  |  |  |  |  |  | | |  | |  | |  | |  | | |  | |  | |  | |  | |  | |  | |  |
|  | Yes |  |  |  |  |  |  |  |  |  |  |  |  |  | | |  | |  | |  | |  | | |  | |  | |  | |  | |  | |  | |  |
| History of previous mental illness | |  |  |  |  |  |  |  |  |  |  |  |  |  | | |  | |  | |  | |  | | |  | |  | |  | |  | |  | |  | |  |
|  | No |  |  |  |  |  |  |  |  |  |  |  |  |  | | |  | |  | |  | |  | | |  | |  | |  | |  | |  | |  | |  |
|  | Yes |  |  |  |  |  |  |  |  |  |  |  |  |  | | |  | |  | |  | |  | | |  | |  | |  | |  | |  | |  | |  |
| Family members with COVID-19 | |  |  |  |  |  |  |  |  |  |  |  |  |  | | |  | |  | |  | |  | | |  | |  | |  | |  | |  | |  | |  |
|  | No | Ref. |  |  | Ref. |  |  | Ref. |  |  | Ref. |  |  |  | | |  | |  | |  | |  | | |  | |  | |  | |  | |  | |  | |  |
|  | Yes | 1.08 | 0.56-2.09 | 0.814 | 1.04 | 0.71-1.52 | 0.856 | 1.82 | 0.49-6.76 | 0.372 | 2.11 | 0.57-7.78 | 0.261 |  | | |  | |  | |  | |  | | |  | |  | |  | |  | |  | |  | |  |
| Family member decesased from COVID-19 | |  |  |  |  |  |  |  |  |  |  |  |  |  | | |  | |  | |  | |  | | |  | |  | |  | |  | |  | |  | |  |
|  | No | Ref. |  |  | Ref. |  |  |  |  |  |  |  |  |  | | |  | |  | |  | |  | | |  | |  | |  | |  | |  | |  | |  |
|  | Yes | 1.10 | 0.43-2.80 | 0.845 | 1.11 | 0.34-3.64 | 0.867 |  |  |  |  |  |  |  | | |  | |  | |  | |  | | |  | |  | |  | |  | |  | |  | |  |
| Economic income in MLW | |  |  |  |  |  |  |  |  |  |  |  |  |  | | |  | |  | |  | |  | | |  | |  | |  | |  | |  | |  | |  |
|  | 1 to 3 | Ref. |  |  | Ref. |  |  |  |  |  |  |  |  |  | | |  | |  | |  | |  | | |  | |  | |  | |  | |  | |  | |  |
|  | 3 to 5 | 1.04 | 0.21-5.14 | 0.966 | 1.15 | 0.16-8.21 | 0.891 |  |  |  |  |  |  |  | | |  | |  | |  | |  | | |  | |  | |  | |  | |  | |  | |  |
|  | 5 to 7 | 0.70 | 0.28-1.65 | 0.393 | 0.65 | 0.38-1.12 | 0.124 |  |  |  |  |  |  |  | | |  | |  | |  | |  | | |  | |  | |  | |  | |  | |  | |  |
|  | 7 to 10 | 1.42 | 0.71-2.82 | 0.319 | 1.36 | 0.47-3.99 | 0.571 |  |  |  |  |  |  |  | | |  | |  | |  | |  | | |  | |  | |  | |  | |  | |  | |  |
|  | > 10 | 0.69 | 0.13-3.71 | 0.664 | 0.67 | 0.06-8.26 | 0.758 |  |  |  |  |  |  |  | | |  | |  | |  | |  | | |  | |  | |  | |  | |  | |  | |  |
| Doctor | |  |  |  |  |  |  |  |  |  |  |  |  |  | | |  | |  | |  | |  | | |  | |  | |  | |  | |  | |  | |  |
|  | No |  |  |  |  |  |  | Ref. |  |  | Ref. |  |  |  | | |  | |  | |  | |  | | |  | |  | |  | |  | |  | |  | |  |
|  | Yes |  |  |  |  |  |  | 0.56 | 0.42-0.74 | **<0.001** | 0.74 | 0.64-0.86 | **<0.001** |  | | |  | |  | |  | |  | | |  | |  | |  | |  | |  | |  | |  |
| Role in service | |  |  |  |  |  |  |  |  |  |  |  |  |  | | |  | |  | |  | |  | | |  | |  | |  | |  | |  | |  | |  |
|  | Leader |  |  |  |  |  |  |  |  |  |  |  |  |  | | |  | |  | |  | |  | | |  | |  | |  | |  | |  | |  | |  |
|  | Leader support |  |  |  |  |  |  |  |  |  |  |  |  |  | | |  | |  | |  | |  | | |  | |  | |  | |  | |  | |  | |  |
|  | Operation staff |  |  |  |  |  |  |  |  |  |  |  |  |  | | |  | |  | |  | |  | | |  | |  | |  | |  | |  | |  | |  |
| Appointed/Fixed term | |  |  |  |  |  |  |  |  |  |  |  |  |  | | |  | |  | |  | |  | | |  | |  | |  | |  | |  | |  | |  |
|  | No |  |  |  |  |  |  |  |  |  |  |  |  |  | | |  | |  | |  | |  | | |  | |  | |  | |  | |  | |  | |  |
|  | Yes |  |  |  |  |  |  |  |  |  |  |  |  |  | | |  | |  | |  | |  | | |  | |  | |  | |  | |  | |  | |  |
| Compliance with confinement measures | |  |  |  |  |  |  |  |  |  |  |  |  |  | | |  | |  | |  | |  | | |  | |  | |  | |  | |  | |  | |  |
|  | Yes |  |  |  |  |  |  |  |  |  |  |  |  |  | | |  | |  | |  | |  | | |  | |  | |  | |  | |  | |  | |  |
|  | No |  |  |  |  |  |  |  |  |  |  |  |  |  | | |  | |  | |  | |  | | |  | |  | |  | |  | |  | |  | |  |
| Compliance with distancing measures | |  |  |  |  |  |  |  |  |  |  |  |  |  | | |  | |  | |  | |  | | |  | |  | |  | |  | |  | |  | |  |
|  | Yes |  |  |  |  |  |  |  |  |  |  |  |  |  | | |  | |  | |  | |  | | |  | |  | |  | |  | |  | |  | |  |
|  | No |  |  |  |  |  |  |  |  |  |  |  |  |  | | |  | |  | |  | |  | | |  | |  | |  | |  | |  | |  | |  |
| Severity of pandemic | |  |  |  |  |  |  |  |  |  |  |  |  |  | | |  | |  | |  | |  | | |  | |  | |  | |  | |  | |  | |  |
|  | Mild/Serious |  |  |  |  |  |  |  |  |  |  |  |  |  | | |  | |  | |  | |  | | |  | |  | |  | |  | |  | |  | |  |
|  | Very serious |  |  |  |  |  |  |  |  |  |  |  |  |  | | |  | |  | |  | |  | | |  | |  | |  | |  | |  | |  | |  |
| Confidence in goverment capacity to manage pandemic | |  |  |  |  |  |  |  |  |  |  |  |  |  | | |  | |  | |  | |  | | |  | |  | |  | |  | |  | |  | |  |
|  | No |  |  |  |  |  |  | Ref. |  |  | Ref. |  |  |  | | |  | |  | |  | |  | | |  | |  | |  | |  | |  | |  | |  |
|  | Yes |  |  |  |  |  |  | 1.67 | 0.90-3.09 | 0.104 | 1.60 | 0.81-3.13 | 0.174 |  | | |  | |  | |  | |  | | |  | |  | |  | |  | |  | |  | |  |
| Experience of mistreatment in health care during COVID-19 | |  |  |  |  |  |  |  |  |  |  |  |  |  | | |  | |  | |  | |  | | |  | |  | |  | |  | |  | |  | |  |
|  | No | Ref. |  |  | Ref. |  |  |  |  |  |  |  |  |  | | |  | |  | |  | |  | | |  | |  | |  | |  | |  | |  | |  |
|  | Yes | 2.80 | 1.69-4.64 | **<0.001** | 2.72 | 1.28-5.79 | **0.009** |  |  |  |  |  |  |  | | |  | |  | |  | |  | | |  | |  | |  | |  | |  | |  | |  |
| Concern about infecting family members | |  |  |  |  |  |  |  |  |  |  |  |  |  | | |  | |  | |  | |  | | |  | |  | |  | |  | |  | |  | |  |
|  | Not at all/ a little |  |  |  |  |  |  |  |  |  |  |  |  |  | | |  | |  | |  | |  | | |  | |  | |  | |  | |  | |  | |  |
|  | Moderate |  |  |  |  |  |  |  |  |  |  |  |  |  | | |  | |  | |  | |  | | |  | |  | |  | |  | |  | |  | |  |
|  | A lot/ Extreme |  |  |  |  |  |  |  |  |  |  |  |  |  | | |  | |  | |  | |  | | |  | |  | |  | |  | |  | |  | |  |
| Concern about becoming infected | |  |  |  |  |  |  |  |  |  |  |  |  |  | | |  | |  | |  | |  | | |  | |  | |  | |  | |  | |  | |  |
|  | Not at all/ a little |  |  |  |  |  |  | Ref. |  |  | Ref. |  |  |  | | |  | |  | |  | |  | | |  | |  | |  | |  | |  | |  | |  |
|  | Moderate |  |  |  |  |  |  | 0.82 | 0.17-4.06 | 0.811 | 0.64 | 9.13-3.13 | 0.581 |  | | |  | |  | |  | |  | | |  | |  | |  | |  | |  | |  | |  |
|  | A lot/ Extreme |  |  |  |  |  |  | 0.65 | 0.33-1.32 | 0.236 | 0.60 | 0.20-1.79 | 0.363 |  | | |  | |  | |  | |  | | |  | |  | |  | |  | |  | |  | |  |
| Concern about availability of PPE | |  |  |  |  |  |  |  |  |  |  |  |  |  | | |  | |  | |  | |  | | |  | |  | |  | |  | |  | |  | |  |
|  | Not at all/ a little | Ref. |  |  | Ref. |  |  |  |  |  |  |  |  |  | | |  | |  | |  | |  | | |  | |  | |  | |  | |  | |  | |  |
|  | Moderate | 0.94 | 0.19-4.68 | 0.941 | 0.94 | 0.25-3.62 | 0.933 |  |  |  |  |  |  |  | | |  | |  | |  | |  | | |  | |  | |  | |  | |  | |  | |  |
|  | A lot/ Extreme | 1.34 | 0.62-2.90 | 0.460 | 1.37 | 0.85-2.22 | 0.198 |  |  |  |  |  |  |  | | |  | |  | |  | |  | | |  | |  | |  | |  | |  | |  | |  |
| Concern about not having health insurance | |  |  |  |  |  |  |  |  |  |  |  |  |  | | |  | |  | |  | |  | | |  | |  | |  | |  | |  | |  | |  |
|  | Not at all/ a little |  |  |  |  |  |  |  |  |  |  |  |  |  | | |  | |  | |  | |  | | |  | |  | |  | |  | |  | |  | |  |
|  | Moderate |  |  |  |  |  |  |  |  |  |  |  |  |  | | |  | |  | |  | |  | | |  | |  | |  | |  | |  | |  | |  |
|  | A lot/ Extreme |  |  |  |  |  |  |  |  |  |  |  |  |  | | |  | |  | |  | |  | | |  | |  | |  | |  | |  | |  | |  |
| Concern about working in a COVID Environment | |  |  |  |  |  |  |  |  |  |  |  |  |  | | |  | |  | |  | |  | | |  | |  | |  | |  | |  | |  | |  |
|  | Not at all/ a little | Ref. |  |  | Ref. |  |  | Ref. |  |  | Ref. |  |  |  | | |  | |  | |  | |  | | |  | |  | |  | |  | |  | |  | |  |
|  | Moderate | 1.77 | 0.57-5.47 | 0.324 | 1.82 | 0.77-4.28 | 0.172 | 3.12 | 1.20-8.07 | **0.019** | 3.62 | 1.80-7.26 | **<0.001** |  | | |  | |  | |  | |  | | |  | |  | |  | |  | |  | |  | |  |
|  | A lot/ Extreme | 1.84 | 0.62-5.41 | 0.270 | 1.82 | 0.53-6.21 | 0.340 | 7.63 | 1.15-50.69 | **0.036** | 8.20 | 0.88-76.80 | 0.065 |  | | |  | |  | |  | |  | | |  | |  | |  | |  | |  | |  | |  |
| Concern about being marginalized by family environment | |  |  |  |  |  |  |  |  |  |  |  |  |  | | |  | |  | |  | |  | | |  | |  | |  | |  | |  | |  | |  |
|  | Not at all/ a little |  |  |  |  |  |  |  |  |  |  |  |  |  | | |  | |  | |  | |  | | |  | |  | |  | |  | |  | |  | |  |
|  | Moderate |  |  |  |  |  |  |  |  |  |  |  |  |  | | |  | |  | |  | |  | | |  | |  | |  | |  | |  | |  | |  |
|  | A lot/ Extreme |  |  |  |  |  |  |  |  |  |  |  |  |  | | |  | |  | |  | |  | | |  | |  | |  | |  | |  | |  | |  |
| Concern about being marginalized by the neighborhood | |  |  |  |  |  |  |  |  |  |  |  |  |  | | |  | |  | |  | |  | | |  | |  | |  | |  | |  | |  | |  |
|  | Not at all/ a little | Ref. |  |  | Ref. |  |  |  |  |  |  |  |  |  | | |  | |  | |  | |  | | |  | |  | |  | |  | |  | |  | |  |
|  | Moderate | 0.66 | 0.45-0.96 | **0.029** | 0.63 | 0.44-0.90 | 0.172 |  |  |  |  |  |  |  | | |  | |  | |  | |  | | |  | |  | |  | |  | |  | |  | |  |
|  | A lot/ Extreme | 1.28 | 1.09-1.52 | 0.004 | 1.45 | 0.53-6.21 | 0.340 |  |  |  |  |  |  |  | | |  | |  | |  | |  | | |  | |  | |  | |  | |  | |  | |  |
| Concern about being an asymptomatic patient | |  |  |  |  |  |  |  |  |  |  |  |  |  | | |  | |  | |  | |  | | |  | |  | |  | |  | |  | |  | |  |
|  | Not at all/ a little | Ref. |  |  | Ref. |  |  |  |  |  |  |  |  |  | | |  | |  | |  | |  | | |  | |  | |  | |  | |  | |  | |  |
|  | Moderate | 0.72 | 0.14-3.81 | 0.700 | 0.69 | 0.16-2.90 | 0.610 |  |  |  |  |  |  |  | | |  | |  | |  | |  | | |  | |  | |  | |  | |  | |  | |  |
|  | A lot/ Extreme | 1.20 | 0.47-3.04 | 0.699 | 1.16 | 0.92-1.46 | 0.203 |  |  |  |  |  |  |  | | |  | |  | |  | |  | | |  | |  | |  | |  | |  | |  | |  |
| Resilience | |  |  |  |  |  |  |  |  |  |  |  |  |  | | |  | |  | |  | |  | | |  | |  | |  | |  | |  | |  | |  |
|  | Low |  |  |  | Ref. |  |  |  |  |  | Ref. |  |  |  | | |  | |  | |  | |  | | |  | |  | |  | |  | |  | |  | |  |
|  | High |  |  |  | 0.89 | 0.66-1.20 | 0.459 |  |  |  | 0.70 | 0.46-1.09 | 0.115 |  | | |  | |  | |  | |  | | |  | |  | |  | |  | |  | |  | |  |
| Insomnia | |  |  |  |  |  |  |  |  |  |  |  |  |  | | |  | |  | |  | |  | | |  | |  | |  | |  | |  | |  | |  |
|  | No |  |  |  | Ref. |  |  | Ref. |  |  | Ref. |  |  |  | | |  | |  | |  | |  | | |  | |  | |  | |  | |  | |  | |  |
|  | Yes |  |  |  | 1.30 | 0.63-2.69 | 0.474 | 2.53 | 1.52-4.22 | **<0.001** | 2.56 | 1.12-5.85 | **0.026** |  | | |  | |  | |  | |  | | |  | |  | |  | |  | |  | |  | |  |
| Physical activity level | |  |  |  |  |  |  |  |  |  |  |  |  |  | | |  | |  | |  | |  | | |  | |  | |  | |  | |  | |  | |  |
|  | Low |  |  |  | Ref. |  |  |  |  |  | Ref. |  |  |  | | |  | |  | |  | |  | | |  | |  | |  | |  | |  | |  | |  |
|  | Moderate |  |  |  | 1.13 | 0.21-5.99 | 0.888 |  |  |  | 0.48 | 0.06-4.06 | 0.503 |  | | |  | |  | |  | |  | | |  | |  | |  | |  | |  | |  | |  |
|  | High |  |  |  | 1.07 | 0.77-1.50 | 0.682 |  |  |  | 1.36 | 0.57-3.23 | 0.482 |  | | |  | |  | |  | |  | | |  | |  | |  | |  | |  | |  | |  |
| Burnout syndrome | |  |  |  |  |  |  |  |  |  |  |  |  |  | | |  | |  | |  | |  | | |  | |  | |  | |  | |  | |  | |  |
|  | No | Ref. |  |  | Ref. |  |  | Ref. |  |  | Ref. |  |  |  | | |  | |  | |  | |  | | |  | |  | |  | |  | |  | |  | |  |
|  | Yes | 5.73 | 1.77-18.48 | **0.004** | 5.56 | 1.93-16.04 | **0.001** | 1.4 | 0.58-3.39 | 0.452 | 1.74 | 1.13-2.68 | **0.012** |  | | |  | |  | |  | |  | | |  | |  | |  | |  | |  | |  | |  |
| Eating disorder | |  |  |  |  |  |  |  |  |  |  |  |  |  | | |  | |  | |  | |  | | |  | |  | |  | |  | |  | |  | |  |
|  | No |  |  |  |  |  |  | Ref. |  |  | Ref. |  |  |  | | |  | |  | |  | |  | | |  | |  | |  | |  | |  | |  | |  |
|  | Yes |  |  |  |  |  |  | 2.3 | 2.17-2.43 | **<0.001** | 2.29 | 1.71-3.07 | **<0.001** |  | | |  | |  | |  | |  | | |  | |  | |  | |  | |  | |  | |  |
| Risk of alcohol consumption | |  |  |  |  |  |  |  |  |  |  |  |  |  | | |  | |  | |  | |  | | |  | |  | |  | |  | |  | |  | |  |
|  | Low | Ref. |  |  | Ref. |  |  |  |  |  |  |  |  |  | | |  | |  | |  | |  | | |  | |  | |  | |  | |  | |  | |  |
|  | Moderate | 4.36 | 0.47-39.98 | 0.193 | 4.20 | 0.47-37.45 | 0.198 |  |  |  |  |  |  |  | | |  | |  | |  | |  | | |  | |  | |  | |  | |  | |  | |  |
| Risk of tobacco use | |  |  |  |  |  |  |  |  |  |  |  |  |  | | |  | |  | |  | |  | | |  | |  | |  | |  | |  | |  | |  |
|  | Low |  |  |  | Ref. |  |  |  |  |  | Ref. |  |  |  | | |  | |  | |  | |  | | |  | |  | |  | |  | |  | |  | |  |
|  | Moderate |  |  |  | 1.66 | 0.48-5.72 | 0.424 |  |  |  | 0.63 | 0.07-5.29 | 0.667 |  | | |  | |  | |  | |  | | |  | |  | |  | |  | |  | |  | |  |
| *p-values ​​obtained with Generalized Linear Models (GLM), Poisson family, log link function, robust variance, hospital as cluster | | | | | | | | | | | | | |  | | |  | |  | |  | |  | | |  | |  | |  | |  | |  | |  | |  |
| *Model 3: Adjusted for variables found statistically significant in Model 2, as well as key theoretically plausible confounders, including age, sex, resilience, insomnia, physical activity level, and tobacco use | | | | | | | | | | | | | | | | |  | |  | |  | |  | | |  | |  | |  | |  | |  | |  | |  |
